# The SINEB1 element in the long non-coding RNA *Malat1* is necessary for TDP-43 proteostasis

**DOI:** 10.1093/nar/gkz1176

**Published:** 2019-12-21

**Authors:** Tuan M Nguyen, Elena B Kabotyanski, Lucas C Reineke, Jiaofang Shao, Feng Xiong, Joo-Hyung Lee, Julien Dubrulle, Hannah Johnson, Fabio Stossi, Phoebe S Tsoi, Kyoung-Jae Choi, Alexander G Ellis, Na Zhao, Jin Cao, Oluwatoyosi Adewunmi, Josephine C Ferreon, Allan Chris M Ferreon, Joel R Neilson, Michael A Mancini, Xi Chen, Jongchan Kim, Li Ma, Wenbo Li, Jeffrey M Rosen

**Affiliations:** 1 Cancer Research Institute, Beth Israel Deaconess Cancer Center, Department of Medicine and Pathology, Beth Israel Deaconess Medical Center, Harvard Medical School, Boston, MA 02115, USA; 2 Department of Molecular and Cellular Biology, Baylor College of Medicine, Houston, TX 77030, USA; 3 Department of Neuroscience, Baylor College of Medicine, Houston, TX 77030, USA; 4 Department of Biochemistry and Molecular Biology, The University of Texas Health Science Center at Houston, McGovern Medical School, Houston, TX 77030, USA; 5 Integrated Microscopy Core, Baylor College of Medicine, Houston, TX 77030, USA; 6 Department of Pharmacology and Chemical Biology, Houston, TX 77030, USA; 7 Michael E. DeBakey High School for Health Professions, Houston, TX 77030, USA; 8 Department of Molecular Physiology and Biophysics, Houston, TX 77030, USA; 9 Department of Experimental Radiation Oncology, The University of Texas MD Anderson Cancer Center, Houston, TX 77030, USA

## Abstract

Transposable elements (TEs) comprise a large proportion of long non-coding RNAs (lncRNAs). Here, we employed CRISPR to delete a short interspersed nuclear element (SINE) in *Malat1*, a cancer-associated lncRNA, to investigate its significance in cellular physiology. We show that *Malat1* with a SINE deletion forms diffuse nuclear speckles and is frequently translocated to the cytoplasm. SINE-deleted cells exhibit an activated unfolded protein response and PKR and markedly increased DNA damage and apoptosis caused by dysregulation of TDP-43 localization and formation of cytotoxic inclusions. TDP-43 binds stronger to *Malat1* without the SINE and is likely ‘hijacked’ by cytoplasmic *Malat1* to the cytoplasm, resulting in the depletion of nuclear TDP-43 and redistribution of TDP-43 binding to repetitive element transcripts and mRNAs encoding mitotic and nuclear-cytoplasmic regulators. The SINE promotes *Malat1* nuclear retention by facilitating *Malat1* binding to HNRNPK, a protein that drives RNA nuclear retention, potentially through direct interactions of the SINE with KHDRBS1 and TRA2A, which bind to HNRNPK. Losing these RNA–protein interactions due to the SINE deletion likely creates more available TDP-43 binding sites on *Malat1* and subsequent TDP-43 aggregation. These results highlight the significance of lncRNA TEs in TDP-43 proteostasis with potential implications in both cancer and neurodegenerative diseases.

## INTRODUCTION

Transposable elements (TEs) are well recognized to be pervasive in mammalian genome yet their roles in gene regulation still remain elusive. TEs comprise 49.9% of the genome with the long interspersed nuclear element (LINE) L1 and SINE Alu families as the most prevalent in the human genome accounting for 29% of genomic sequences (1). Eighty three percent of long non-coding RNAs (lncRNAs) contain TEs, which comprise 42% of lncRNA sequences, whereas only 6.2% of protein-coding genes contain TEs, which comprise only 0.32% of their nucleotides (nts) (1). The relatively large contribution of TEs to the composition of lncRNAs suggest that they are more robust against natural selection through evolution as components of lncRNAs as compared with those of protein-coding genes. Thus, TEs may play a significant role in maintaining the proper regulatory functions of lncRNAs. In fact, TEs in lncRNAs have been postulated to regulate mRNA decay (2), mRNA translation (3) and chromatin remodeling (4–6). A point mutation in a TE of a novel lncRNA *SLC7A2-IT1A/B* has even been associated with lethal encephalopathy (7). Despite these findings, the effect of the loss of a TE in a lncRNA on its endogenous expression and resulting cellular physiology has not been investigated. We show that the deletion of a SINEB1 in the murine lncRNA *Malat1* causes activation of a global unfolded protein response (UPR) and has detrimental effects on cell survival, genomic stability and cell cycle progression. The latter subsequent effects are induced by cytoplasmic export of *Malat1*, due to the SINE deletion, together with its direct binding partner, TDP-43. TDP-43 is a predominantly nuclear protein essential for RNA metabolism, which displays increased binding to *Malat1* in the absence of the SINE and forms cytotoxic inclusions. Cytoplasmic translocation of TDP-43 also depletes nuclear TDP-43, reprogramming TDP-43 binding to mRNAs of cell cycle and nuclear-cytoplasmic regulators and potentially causing defects in their processing and function. The SINE promotes nuclear retention by facilitating *Malat1* binding to HNRNPK, a RNA-binding protein (RBP) known to drive RNA nuclear retention, potentially through direct interactions of the SINE with KHDRBS1 and TRA2A, which interact with HNRNPK. The loss of these RNA–protein interactions due to the SINE deletion may create more available TDP-43 binding sites on *Malat1* and subsequent TDP-43 mis-localization and aggregation.

## MATERIALS AND METHODS

### Cell lines

HC11 cells (mammary epithelium, ATCC CRL-3062) were grown in RPMI-1640 with l-glutamine and HEPES (GenDEPOT, CM058), 10% (v/v) fetal bovine serum (FBS) (GenDEPOT, F0900-050), 5 μg/ml insulin (MilliporeSigma, I5500), 10 ng/ml epidermal growth factor (MilliporeSigma, EA140) and 1 U/ml Antibiotic-Antimycotic (ThermoFisher Scientific, 15240062). 293T cells (ATCC CRL-3216) were cultured in DMEM (GenDEPOT, CM002-310), 10% (v/v) FBS (GenDEPOT, F0900-050) and 1 U/ml Antibiotic-Antimycotic (ThermoFisher Scientific, 15240062).

### Plasmids

Plasmids pSpCas9(BB)-2A-GFP (PX458) (Addgene, 48138) and pSpCas9(BB)-2A-Puro (PX459) V2.0 (Addgene, 62988) were used for generation of SINE- and CER-deleted cells. Fucci reporters mCherry-hCdt1(30/120)/pCSII-EF-MCS (mCherry-Cdt) and AmCyan-hGeminin(1/110)/pCSII-EF-MCS (AmCyan-Geminin) were provided by Dr Atsushi Miyawaki (RIKEN) through a material transfer agreement. Plasmids psPAX2 (Addgene, 12260), pMD2.G (Addgene, 12259) and pLKO.1 (Addgene, 8453) were used for lentivirus production and generation of stable cell lines expressing Fucci reporters and shRNAs. Plasmid pcDNA3 TDP-43-eGFP full length used for live imaging of TDP-43 localization and FRAP was generated by subcloning TDP-43 DNA sequence from pDuet TDP-43 WT plasmid (Addgene, 27462) into pcDNA3-EGFP vector (Addgene plasmid #13031) using Gibson Assembly Master Mix (NEB, E2611S). Plasmids pcDNA3.1 WT *Malat1* and pcDNA3.1 SINE used for rescue experiments in ΔSINE cells were generated by subcloning full length WT mouse *Malat1* cDNA and the mouse *Malat1* SINEB1 alone into pcDNA3.1/Hygro(+) (ThermoFisher Scientific, V87020), respectively.

### Generation of SINE and CER deletion with CRISPR

**Figure 1.**
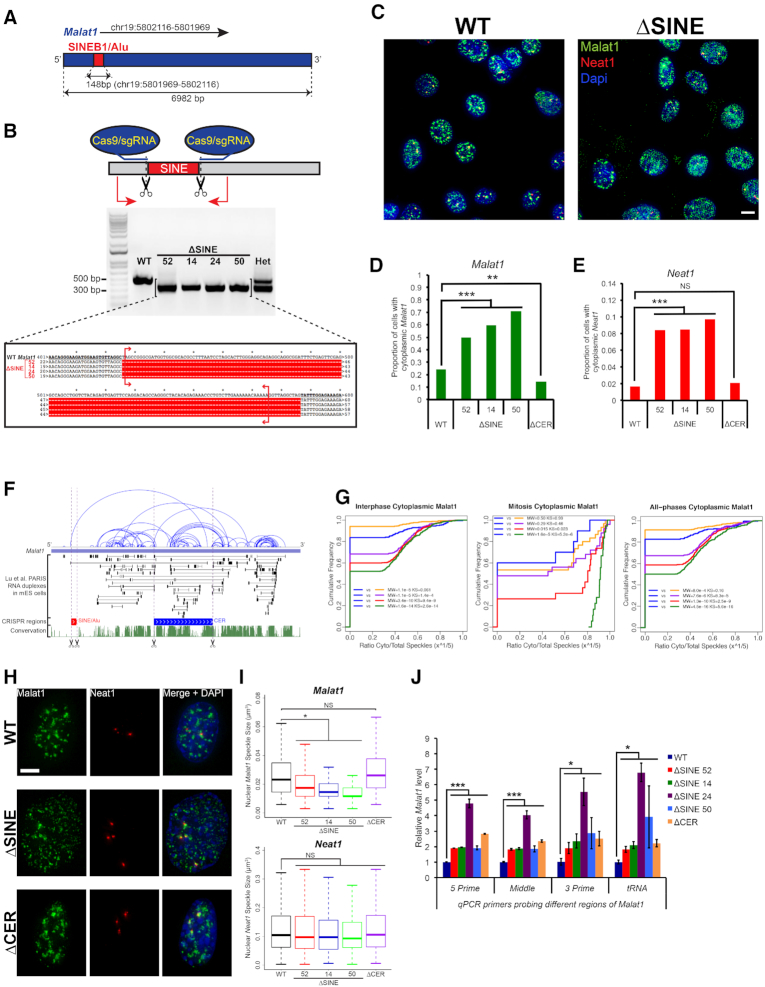
*Malat1* ΔSINE exhibits increased cytoplasmic localization and forms diffuse nuclear speckles. (**A**) Relative position of the SINE within the genomic locus of *Malat1*. (**B**) Generation and validation of ΔSINE cells. Upper: Schema of CRISPR strategy for deletion of the SINE. Middle: Gel electrophoresis image screening for clones with complete SINE deletion. Genomic DNA isolated from each clone was amplified with PCR using primers (red arrows) flanking the deleted region. The upper band is approximately 150-bp larger than the lower band, which corresponds with the length of the SINE. WT, wildtype; Het, heterozygous clone with both WT and SINE-deleted *Malat1*. Lower: Alignment of Sanger sequencing reads confirming complete SINE deletion. Red brackets denote the exact location of the SINE. (**C**) Representative *Malat1* and *Neat1* fluorescent *in situ* hybridization (FISH) images of WT and ΔSINE cells (scale bar = 10 μm). Note the prevalence of cytoplasmic *Malat1* speckles and their diffuse pattern in ΔSINE cells without mitotic features. (**D** and **E**) Quantification of cells with cytoplasmic *Malat1* (upper) or *Neat* (lower) speckles, respectively. *Pearson's* chi-squared test was used for comparisons. ***P < 0.001; *n* ≈ 365 cells for each group. (**F**) CER, but not SINE is involved in formation of intramolecular duplexes. Sequences of PARIS RNA duplexes extracted from Lu *et al.* (14) were aligned to *Malat1*’s sequence. Thin black lines and blue arcs connect regions that form duplexes as emphasized in black boxes. Purple dashed lines indicate locations of the SINE (red) and the CER (blue) relative to the duplexes. Scissors symbols demarcate regions deleted with CRISPR. Green track illustrates phastCons conservation score across 60 vertebrates. (**G**) Interphase ΔSINE cells have significantly more cytoplasmic *Malat1* than interphase WT cells. Cumulative distribution of the ratio of cytoplasmic over total Malat1 speckle number in interphase (left), mitotic (middle), and all-phases (right) cells. Interphase and mitotic cells were classified based on DAPI signal intensity and texture, using a custom-made MATLAB script. WT, ΔSINE52, ΔSINE14, ΔSINE50 and ΔCER are shown in blue, purple, red, green, and orange, respectively. The cytoplasmic *Malat1* ratio was transformed with *x*^1/5^, where *x* is the ratio, to approximate normalization for non-normally distributed data. Two different non-parametric tests, Mann–Whitney *U* test (MW) and Kolmogorov–Smirnov test (KS), were used showing comparable *P* values. Note that although more cytoplasmic Malat1 is detected in both interphase and mitotic ΔSINE cells, more significant *P* values are observed for interphase cells. (**H**) *Malat1* ΔSINE forms diffuse nuclear speckles (scale bar = 5 μm). Shown are representative FISH images of ≈200 cells for each group. (**I**) *Malat1* nuclear speckles are smaller in ΔSINE cells but not *Neat1*. Box plots comparing *Malat1* and *Neat1* speckle sizes across cell groups. Speckle sizes were quantified using a custom-made MATLAB script as described in Materials and Methods. *P < 0.05; NS, not significant (*P* > 0.05); *n* ≈ 365 cells for each group. (**J**) *Malat1* expression increases in the absence of the SINE. Comparison of *Malat1* RNA levels between ΔSINE and ΔCER versus WT cells. RNAs isolated from the cells were analyzed with qPCR using primers probing four different regions of *Malat1* from 5′ to 3′. *P < 0.05; ***P < 0.05; *n* = 3 biological replicates.

As described in Figure 1B and Supplementary Figure S1B, pairs of single guide RNAs (sgRNAs) flanking the SINE or the CER were designed and cloned into PX458 and PX459 plasmids as previously described (8). Custom sgRNA sequences found in Supplementary Table S2 were purchased from Integrated DNA Technologies (IDT). The PX458 and PX459 plasmids carrying sgRNA pairs were then transfected in a 1:1 ratio to HC11 cells grown to 70–80% confluence using Lipofectamine 3000 Transfection Reagent (ThermoFisher Scientific, L3000015) following the manufacturer's instructions. After 48 h, transfected cells were sorted with flow cytometry for strong GFP-positive cells, which were then seeded as single cells per well in a 96-well plate with conditioned media. Genomic DNA from clones of the single cells were isolated for PCR genotyping to identify clones with complete SINE or CER deletion using DreamTaq Green PCR Master Mix (ThermoFisher Scientific, K1082). Primer sequences for the PCR screen purchased from IDT were designed as described in Figure 1B and Supplementary Figure S1B and can be found in Supplementary Table S1. The majority of clones were heterozygous for the deletion with an upper band (WT) and a lower band (CRISPR mutant). Clones with complete deletions had only the lower band and were expanded and frozen down at early passages using the freezing media recommended by ATCC for HC11 cells.

### smFISH analysis

WT and ΔSINE- and ΔCER- cells grown on glass coverslips (Corning, 354087) were fixed in 4% purified paraformaldehyde (Electron Microscopy Sciences, RT 15710) in ribonuclease (RNase)-free PBS for 15 min and then permeabilized with 70% (v/v) ethanol in RNase-free water at 4°C for a minimum of 1 h. Cells were washed in 1 ml of wash buffer (2× saline sodium citrate [SSC] plus 10% formamide) followed by overnight hybridization with 40 nM RNA FISH probes at 37°C in Stellaris RNA FISH Hybridization Buffer (LGC Biosearch Technologies, SMF-HB1-10), followed by one change in wash buffer (30 min at 37°C), and a second wash in buffer containing 1 μg/ml DAPI (MilliporeSigma, D8417) (10 min at 37°C) as previously described in Orjalo *et al.* (9). Vectashield (Vector Laboratories, H-1000-10) was used as mounting medium. RNA FISH probes used were *Malat1* (LGC Biosearch Technologies, SMF-3008-1) and *Neat1* (LGC Biosearch Technologies, VSMF-3031-5). High quality/high resolution imaging was performed on a GE Healthcare DVLive epifluorescence image restoration microscope using an Olympus PlanApo 100×/1.4 NA objective and a 1.9k × 1.9k sCMOS camera. *Z* stacks (0.25 μm) covering the whole nucleus (∼10 μm) were acquired before applying a conservative restorative algorithm for quantitative image deconvolution. Max intensity projections were generated for illustration purposes only.

### Image analysis for smFISH speckles, nucleus and micronuclei quantification

*Malat1* and *Neat1* speckles counting was performed using a custom-made MATLAB script (https://github.com/juldub07/MALAT). Briefly, nuclei were segmented from the *z*-projected DAPI image stack using a watershed algorithm, and segmented nuclei were further classified as interphase or mitotic, based upon DAPI signal intensity and texture. The whole cell area was then approximated by tessellation of the nuclear compartment, and the cytoplasmic compartment was determined by subtracting the nuclear compartment from the whole cell compartment. *Malat1* and *Neat* speckle counting were done in three dimensions: after local background correction, each *z*-stack from the *Malat1* and *Neat1* channels was binarized using the Otsu method and the number, size and mean fluorescent intensity of three-dimensional objects, defined as 26-connected neighborhood components, were calculated for the nucleus and cytoplasmic compartments of each cell, respectively. Cells with micronuclei based on the DAPI signal were counted in at least ten random images per sample.

### Immunofluorescence

Cells were grown to log phase on glass coverslips (Corning, 354087), washed twice with PBS, then fixed in 4% (w/v) paraformaldehyde in PBS for 15 min at RT. Fixed cells were then washed thrice in PBS for 5 min each and permeabilized with 0.5% (v/v) Triton-X in PBS (PBST) for 30 min. Permeabilized cells were washed thrice with PBS for 5 min each then blocked in 3% (w/v) bovine serum albumin (BSA) diluted in PBST for 1 h. After blocking, cells were incubated with the primary antibody p-H3 (Ser^10^) (MilliporeSigma, 06570) diluted 1:250 in PBST with 3% BSA overnight at 4°C. Goat anti-Rabbit IgG (H+L) Cross-Adsorbed Secondary Antibody, Alexa Flour 594 (ThermoFisher Scientific, A-11012) was used according to the manufacturer's instructions following primary antibody incubation and three washes with PBST. Stained cells were washed three times in PBS, incubated with 1 drop of DAPI (ThermoFisher Scientific, R37606) in 1ml PBS for 10 min then mounted on a glass slide with Aqua-Poly/Mount (18606-20). An Olympus BX50 fluorescence microscope was used to image the samples at 20–40×. Images were subsequently merged and analyzed with ImageJ 1.48v. p-H3-positive cells and cells with aggregated nuclei were counted in at least 10 random images per sample.

### Transmission electron microscopy analysis

WT, ΔSINE and ΔCER cells grown to log phase in a six-well plate (VWR, 10062-892) were fixed with a solution containing 3% glutaraldehyde plus 2% paraformaldehyde in 0.1 M cacodylate buffer, pH 7.3, then washed in 0.1 M sodium cacodylate buffer and treated with 0.1% Millipore-filtered cacodylate buffered tannic acid, postfixed with 1% buffered osmium, and stained en bloc with 1% Millipore-filtered uranyl acetate. The samples were dehydrated in increasing concentrations of ethanol, infiltrated, and embedded in LX-112 medium. The samples were polymerized in a 60°C oven for ∼3 days. Ultrathin sections were cut in a Leica Ultracut Microtome (Leica Microsystems), stained with uranyl acetate and lead citrate in a Leica EM Stainer, and examined in a JEM-1010 transmission electron microscope (JEOL USA, Inc.) at an accelerating voltage of 80 kV. Digital images were obtained using AMT Imaging System (Advanced Microscopy Techniques).

### Chemical treatments

Cells were treated with Tunicamycin (MilliporeSigma, 474791) or DMSO for 24 h, and collected for RNA and protein isolation following each treatment. For caspase inhibition, cells were treated with 50 μM of pan-caspase inhibitor Z-VAD-FMK (Cayman Chemical, 14463) or DMSO for 24 h, then collected for western blot analysis.

### qPCR analysis

RNA processing and qPCR analysis were carried out exactly as previously described in Nguyen *et al.* (10). Primers used are listed in Supplementary Table S1. RNA levels were normalized to GAPDH or HPRT values.

### Western blot analysis

For total protein extraction, HC11 cells grown to log phase were collected and lysed in ice-cold RIPA Lysis and Extraction Buffer (ThermoFisher Scientific, 89901) with freshly added phosphatase (Sigma-Aldrich, 04906837001) and protease (Sigma-Aldrich, 04693124001) inhibitors following the manufacturer's instructions. Cells were incubated in RIPA buffer with constant rotation for 1 h at 4°C, and cell lysates were collected by centrifugation at maximum speed (∼14 000 rpm) for 20 min at 4°C. Twenty to fifty microgram of proteins were fractionated by sodium dodecyl sulfate-polyacrylamide gel electrophoresis (SDS-PAGE) then transferred onto Immobilon-P PVDF Membranes (Millipore, IPVH00010). Membranes were blocked in 3% (w/v) BSA in tris-buffered saline with 1% (v/v) Tween 20 (TBST) for 1 h at RT then incubated overnight at 4°C with the following primary antibodies in 1:1000 dilution: eIF2α (D7D3) (Cell Signaling, 5324S), phospho-eIF2α (Ser^51^) (Cell Signaling, 3597S), γ-H2AX (BioLegend, 613402), Caspase-3 (Cell Signaling, 9662S), cleaved Caspase-3 (ASP^175^) (Cell Signaling, 9661S), PARP (Cell Signaling, 9542S), cleaved PARP (ASP^214^) (Cell Signaling, 9544T), BCL-2 (BD Biosciences, 554218), PERK (C33E10) (Cell Signaling, 3192S), PKR (B-10) (Santa Cruz Biotechnology, sc-6282), TDP-43 (Proteintech, 10782-2-AP), TDP-43 (3H8) (Abcam, ab104223), HDAC2 (Cell Signaling, 2540S), HSP90 (Cell Signaling, 4874S) Vinculin (MilliporeSigma, V9131), GAPDH (Cell Signaling, 2118). Western blot detection was performed using Amersham Imager 600 imager (GE Healthcare Life Sciences). Quantification of western blot bands’ relative area and density values were carried out using ImageJ 1.48v (11) following ImageJ User Guide for gel analysis (https://imagej.nih.gov/ij/docs/guide/). Quantified values were normalized by values for loading controls such as Vinculin and GAPDH. For phosphorylated and cleaved proteins, quantified values were normalized by values for both corresponding total proteins and loading controls. For p-PKR detection, immunoprecipitation was performed as previously described (12) using PKR (B-10) antibody (Santa Cruz Biotechnology, sc-6282), followed by western blot analysis for p-PKR using p-PKR (Thr^451^) antibody (ThermoFisher Scientific, 44-668G).

### Cell fractionation

For RIPA/urea fractionation of soluble and insoluble cellular fractions, HC11 cells grown to log phase were collected and resuspended in ice-cold RIPA buffer (ThermoFisher Scientific, 89901) with freshly added phosphatase (Sigma-Aldrich, 04906837001) and protease (Sigma-Aldrich, 04693124001) inhibitors following the manufacturer's instructions. Cells were then sonicated with maximum power for 10 s using a Bioruptor ultrasonicator (Diagenode) and incubated in RIPA buffer with constant rotation for 1 h at 4°C. After that, samples were centrifuged at maximum speed for 30 min at 4°C. The supernatant was collected as the soluble fraction and kept on ice for western blot analysis. The resulting pellet was resuspended in RIPA buffer, re-sonicated and re-centrifuged as shown above twice to completely lyse all soluble cellular contents. The remaining pellet was resuspended in urea buffer (7 M urea, 2 M thiourea, 4% (w/v) CHAPS, 30 mM Tris, pH 8.5), sonicated as shown above and incubated with constant rotation for 1 h at 4°C to completely dissolve all insoluble fractions. The supernatant obtained after 30 min maximum speed centrifugation at 4°C is the insoluble fraction that was used for western blot analysis.

For nuclear/cytoplasmic fractionation, nuclear and cytoplasmic fractions of exponentially growing cells were collected for western blot analysis using the NE-PER Nuclear and Cytoplasmic Extraction Reagents (ThermoFisher Scientific, 78835) following the manufacturer's guideline.

### Polysome analysis

WT and ΔSINE cells grown to 70–80% confluence were collected and process for polysome fractionation as previously described in Nguyen *et al.* (10).

### XBP1 splicing assay

Primers were designed to amplify a cDNA fragment of XBP1 that harbors a splice junction between exon 3 and exon 4. The intronic region connecting exon 3 and exon 4 is amplified in XBP1u transcripts, whereas the region is absent in XBP1s transcripts due to a splicing event induced by ER stress (13). Primers used are listed in Supplementary Table S1. PCR amplified bands visualized by gel electrophoresis were quantified with ImageJ 1.48v as described in the western blot analysis section.

### Crystal violet assay

WT, ΔSINE and ΔCER cells were seeded at 200 000 cells per well in a six-well plate (VWR, 10062-892). Cells were washed once with PBS and fixed in 4% (w/v) paraformaldehyde in PBS for 15 min after 1 day, 2 days and 3 days post-seeding. Fixed cells were then washed once with water, stained with 0.1% (w/v) crystal violet (MilliporeSigma, C3886) in water with 10% (v/v) ethanol for 20 min. Stained cells were washed thrice with water and air dried overnight. The dry crystal violet stain was dissolved in 10% (v/v) acetic acid for 10 min with gentle shaking, then diluted 1:4 in water for absorbance measurement at a wavelength of 590 nm using Synergy HTX Multi-Mode Reader (BioTek Instruments, Inc.). Proliferation curves were generated based on changes in absorbance values relative to day 1.

### Overexpression of TDP-43-GFP and live imaging

The pcDNA3 TDP-43-eGFP full length plasmid was transfected into WT and ΔSINE cells at ∼40% confluence seeded in a low-wall 35-mm imaging dish (Ibidi, 80136) using Lipofectamine 3000 Transfection Reagent (ThermoFisher Scientific, L3000015) following the manufacturer's instructions. After 24–48 h, cells were added with two drops of Nuc-Blue Live ReadyProbes Reaget (ThermoFisher Scientific, R37605) and imaged using EVOS FL Cell Imaging System (ThermoFisher Scientific) at 40–60×. Images were subsequently merged and analyzed with ImageJ 1.48v. Quantification of cells with different TDP-43-GFP patterns was obtained by counting at least 10 random images per sample.

### Fluorescence recovery after photobleaching (FRAP) imaging

FRAP imaging of cells, 2 days after transfection with the pcDNA3 TDP-43-eGFP full length plasmid, was performed on a Zeiss LSM780 laser-scanning confocal microscope system at 37°C and 5% CO_2_. Different nuclear and cytoplasmic regions of interest (ROIs; 5–8 nuclear or cytoplasmic regions in 3–5 cells) with ∼0.5–2 μm diameter circles were chosen for the analysis. Reference ROIs were drawn in adjacent regions. Following 2–3 baseline images, ROIs were bleached for 10–20 s at 100% laser power (488 nm laser, 33 μW power measured at the objective), and were imaged for up to 5 min post-bleaching to check for fluorescence recovery. FRAP recovery curves were corrected for background photobleaching normalized to pre-bleach intensity values.

### Generation of ΔSINE cells with overexpression of WT *Malat1* and the SINE alone

ΔSINE cells, transfected with pcDNA3.1 WT *Malat1* or pcDNA3.1 SINE plasmid for 48 hrs, were selected with hygromycin B (Mirus, MIR5930) for stable clones. The stable clones were subsequently analyzed with qPCR for overexpression of WT *Malat1* or the SINE slone using primers that are specific to the constructs as well as western blot analysis.

### Cell cycle analysis with flow cytometry

Collected cells were fixed in 70% (v/v) ethanol overnight at 4°C. Cells were subsequently washed three times with 1× TF Perm/Wash Buffer (BD Biosciences, 51-9008102) and then stained in 1× TF Perm/Wash Buffer with 50 μg/ml propidium iodide (Sigma, P4864) and 0.1 mg/ml RNase A (Sigma-Aldrich, 10109142001). Cell cycle data was acquired using LSR II cytometer (BD Biosciences) and analyzed with FlowJo (v.10.1).

### Lentivirus production for shRNA knockdown

Custom PERK, PKR and Scrb shRNA oligos purchased from MilliporeSigma were cloned into PLKO.1 lentiviral plasmid (Addgene, 8453). TDP-43 shRNA (V3SM11241-231478494) and a Scrb shRNA on the same lentiviral plasmid backbone (VSC11715) were purchased from Dharmacon. Targeted sequences of these shRNAs are listed in Supplementary Table S3. Viral packaging plasmids psPAX2 (Addgene, 12260) and pMD2.G (Addgene, 12259) together with lentiviral shRNA plasmids were transfected to 293T cells in a 2:1:3 ratio using TransIT-293 Transfection Reagent (Mirus, MIR2705) or Lipofectamine 3000 Transfection Reagent (ThermoFisher Scientific, L3000015) following the manufacturer's guideline. Lentiviruses were collected 48 and 72 h after transfection and filtered with 0.45-μm filters. For transduction in HC11 cells, the viral supernatant was mixed with HC11 culture media in a 1:2 ratio then selected with puromycin (for PERK and PKR shRNAs) or sorted for strong RFP-positive cells (for TDP-43 shRNAs) after at least 48 h of transduction.

### Fucci analysis

Lentiviral Fucci reporters mCherry-Cdt and AmCyan-Geminin acquired from Dr Atsushi Miyawaki (RIKEN) were packaged into lentiviruses as described in the lentivirus production for shRNA knockdown section. WT and ΔSINE cells were first transduced with mCherry-Cdt lentiviruses. Strong mCherry-positive cells were then enriched with flow cytometry and further transduced with AmCyan-Geminin lentiviruses. Strong Amcyan-positive cells were collected with flow cytometry and seeded into a 96-well plate for live-cell imaging every 30 min for 72 h using The IncuCyte S3 Live-Cell Analysis System (Essen BioScience). Videos and images were analyzed using the IncuCyte S3 software and ImageJ 1.48v. The videos were used for tracking the length of time each cell stays in mCherry(RFP) or AmCyan(GFP) as well as tracking cells with mitotic catastrophe, in which daughter cells shrink in size and cannot divide further. Relative fluorescent intensity of each color for individual cells during the time-course of one cell cycle was collected from individual images using the IncuCyte S3 software.

### Alignment of PARIS RNA duplexes and previously studied fragments of *Malat1* with the SINE and the CER

For PARIS RNA duplex alignment, genomic coordinates of regions that form RNA duplexes on *Malat1* generated in Lu *et al.* (14) for mouse embryonic stem cells (mES) were used to generate a new bed file that was input into the UCSC genome browser in order to create a custom track with alignment of the duplexes within *Malat1*’s genomic locus. Arcs connecting duplexed regions were generated using IGV_2.4.10. A separate bed file with coordinates of the SINE and the CER were also generated and input into the UCSC genome browser to align with regions that form RNA duplexes on *Malat1*. PhastCons conservation track was enabled within the UCSC genome browser to display conserved elements across the sequence of *Malat1*. For alignment of fragments previously studied in Miygawa *et al.* (15), genomic coordinates of fragments C, E, H, I, J, K and M in the study were converted from human *Malat1* to mouse *Malat1* using the UCSC liftOver tool. A bed file was generated for the coordinates to generate a custom track on the UCSC genomic browser for alignment with the SINE and the CER within the genomic locus of *Malat1*.

### eCLIP-seq

eCLIP-seq was performed following a published protocol (16). Briefly, ∼15 million HC11 cells (WT or *Malat1* mutants) were cross-linked with UV (254 nm, 400 mJ/cm^2^), followed by lysis in 1 ml of iCLIP lysis buffer, digestion with RNase I (ThermoFisher Scientific, AM2295), and then subjected to immunoprecipitation with 8 μg of a high quality TDP-43 antibody (Proteintech, 10782-2-AP) pre-bound to Dynabeads M-280 Sheep Anti-Rabbit IgG (ThermoFisher Scientific, 11204D). After stringent washes (with 1 M NaCl), the TDP-43-RNA complexes were subjected to FastAP (ThermoFisher Scientific, EF0652) and T4 Polynucleotide Kinase (NEB, M0201L) treatment, and then ligated to a 3′ end RNA adaptor, RiL19 (Supplementary Table S4) using on-bead ligation with T4 RNA ligase 1 (NEB, M0437M). The TDP-43-RNA complexes from multiple cell lines were then resolved on a NuPage Bis–Tris gel (ThermoFisher Scientific), and transferred to Nitrocellulous membrane. The region of 35–110 kDa was cut from the membrane, which contains the smaller isoform of TDP-43 and its RNA interactome of ∼220-nt long (∼75 kDa larger than TDP-43 itself), and was then subjected to RNA purification using proteinase K (NEB, P8107S) digestion and phenol-chloroform purification. Purified RNAs were reverse-transcribed using AffinityScript (Agilent, 600107). The rand103Tr3 DNA adapter, containing a random-mer of 10 nucleotides (Supplementary Table S4), was then ligated to the cDNA fragment using T4 RNA Ligase 1 (NEB, M0437M).

The same molecular weight (35–110 kDa) Input RNA (SMInput) was also excised from nitrocellulose membrane. The recovered RNAs were end-repaired by FastAP (ThermoFisher Scientific, EF0652) and T4 Polynucleotide Kinase (NEB, M0201L), and then first ligated to RiL19 RNA adaptor, and then to rand103Tr3, similar to the preparation of immunoprecipitated RNAs for sequencing libraries. The final cDNA library was amplified with two oligos, with 6- or 8-nt barcodes in the reverse oligo, for example, PCR_F_D501 and PCR_R_D701 (Supplementary Table S4), using Q5 High-Fidelity 2× Master Mix (NEB, M0492L). PCR cycles were ∼14–16 for TDP-43 IP groups and ∼8–10 for SMInput groups. The eCLIP libraries were sequenced on a Next-Seq500 platform with a pair-ended 80-nt run mode.

### eCLIP-seq data processing and peaks annotation

The eCLIP-seq data was processed as previously described (16). Briefly, adapters were trimmed by cutadapt (v 1.15) from the raw sequencing reads. The clean reads were then mapped to mouse genome (mm10) with STAR (v 2.5.3a_modified), and PCR duplicates were removed based on random-mer by gscripts pipeline from YeoLab. Average reads derived per sample were ∼25 million, and 8 million reads were used for downstream analysis. Peaks were called by CLIPper with options ‘–bonferroni –superlocal –threshold-method binomial –binomial = 0.05’ using usable Read2. The eCLIP peaks were further normalized against SMInput, and only those significant peaks (Fisher exact test *P*-value < 0.001 and fold-enrichment > 2) were kept. For peaks annotation, the gene regions for significant peaks from each replicate were annotated by HOMER (17). The gene ontology and pathway enrichment analysis were performed by Metascape (http://metascape.org) (18). Over-representation analysis of genes involved in in cell cycle and cellular transport regulation were carried out using ConsensusPathDB’s databases (19). The raw eCLIP-seq data have been deposited to the NCBI Gene Expression Omnibus (GEO; https://www.ncbi.nlm.nih.gov/geo/) under accession number GSE141303.

### Motif enrichment analysis for TDP-43 binding sites

DREME (v.4.12.0) was used to find motifs in all peak sets derived from TDP-43 eCLIP-seq.

### Repetitive element analysis for TDP-43 eCLIP

Trimmed reads were mapped to mouse repetitive elements from RepBase (Version 23.01) using STAR, and the mapped reads were counted by ‘count_aligned_from_sam.py’ from YeoLab pipeline. Unmapped reads were then mapped to the mouse genome (mm10) with the same method. The PCR duplicates were removed based on the UMI. For each repetitive element, the binding affinity was defined as the fold (i.e. RPMfold) for the normalized reads between IP and the corresponding SMInput using R. Repetitive elements with binding affinity >1 in at least one of the three samples were used for subsequent analyses.

### RNA immunoprecipitation (RIP) assay

RIP was performed as previously described (20) using 5 μg of antibodies against HNRNPK (Santa Cruz Biotechnology, sc-28380) and IgG (Santa Cruz Biotechnology, sc-2025). Immunoprecipitated RNA was amplified with RT-PCR using *Malat1* primers then visualized with gel electrophoresis. Relative enrichment was quantified by normalizing HNRNPK IP over IgG IP gel band intensity, which was quantified by ImageJ 1.48v

### Double-stranded RNA quantification

Exponentially growing cells were fixed in 4% paraformaldehyde and subsequently stained with anti-dsRNA antibody (English and Scientific Consulting Kft., K1) in the presence of 0.1% saponin to allow for cell permeabilization. Cells were then stained with Goat anti-Mouse IgG (H+L) Cross-Adsorbed Secondary Antibody, Alexa Flour 594 (ThermoFisher Scientific, A-11005) and analyzed by flow cytometry.

### Correlation analysis for the prevalence of the SINEB1 in mRNAs with increased nuclear accumulation

Genomic coordinates for all copies of the *Malat1* SINEB1 across the mouse genome extracted from the mm10 repeatmasker UCSC database were intersected with coordinates of exons from the mRNAs with available nuclear and cytoplasmic copy numbers previously reported for mouse liver and pancreatic beta cells (21). The SINEB1 copies that overlap with the exons of the mRNAs were further classified into sense (i.e. same strand) and antisense (i.e. opposite strand) SINEB1s based on their strandedness relative to the exons. mRNAs with no exonic overlap with any copies of the SINEB1 are considered to have no SINEB1. Average nuclear/cytoplasmic copy number ratios from biological duplicates for each mRNA reported in Bahar Halpern *et al.* (21) was used for the analysis.

### *Malat1* SINEB1 and CER alignment across species

For *Malat1* SINEB1 RNA alignment, available *Malat1* RNA sequences from other species with alignment to the SINE region of the mouse *Malat1*, extracted from the UCSC genome browser ‘Other mRNAs’ track, were aligned together with the human and mouse *Malat1* RNA sequences using the MUSCLE algorithm (22). The alignment was then visualized with Jalview (23). For DNA alignment of the SINEB1 and the CER, the mouse genomic regions were aligned across 56 eutharian mammals using the ‘Genomic alignments’ function in the Ensembl 92 genome database.

### Identification of RBPs with the highest number of eCLIP peaks overlapping with the SINE-aligned region in human *Malat1*

All significant eCLIP peaks for 120 RBPs in K562 cells were extracted from the ENCODE database in bed format as biological replicates for each RBP, and then overlapped with the human SINEB1-aligned region. Note that K562 cells have the most number of eCLIP datasets deposited into the ENCODE database. Top 10 RBPs with the highest number of SINEB1-overlapping eCLIP peaks, calculated as average peak number of biological replicates, was used for protein-protein network analysis. eCLIP read density profiles across *Malat1* genomic locus was visualized on the UCSC genome browser using BigWig files downloaded from the ENCODE database for RBPs of interest.

### Protein-protein interaction network analysis

STRING 11.0 database was used to identify direct interactions experimentally determined for RBPs of interest.

### Statistical analysis

Statistical tests with suitable underlying assumptions on variance characteristics and data distribution were employed. Unless otherwise noted, two-tailed Student's *t*-tests were used for comparisons between groups.

## RESULTS

### Generation of cells with deletion of the SINEB1 in *Malat1*

*Malat1* is an evolutionarily conserved and abundant lncRNA that has been associated with breast cancer progression and metastasis. *Malat1* on chromosome (chr) 19 of the mouse genome localizes to nuclear speckles (24) and harbors a SINEB1 element near the 5′ end of its 6982-base pair (bp) transcript (Figure 1A). The 148-bp SINEB1 element is a rodent equivalent of primate Alu derived from 7SLRNA through evolution (25). Since Alu elements at the 3′ untranslated regions (UTRs) of mRNAs have previously been implicated in the regulation of mitosis (26), we hypothesized that the SINEB1 in *Malat1* also might play a functional role in cellular physiology. We employed CRISPR-Cas9 technology to delete the SINE of endogenous *Malat1* in a mammary epithelial cell line HC11 using two single guide RNAs (sgRNAs) closely flanking the 5′ and 3′ ends of the SINE within *Malat1* (Figure 1B, Materials and Methods). Four independent clones with complete homozygous SINE deletion (ΔSINE) were generated and validated with Sanger sequencing (Figure 1B).

### *Malat1* without the SINEB1 shuttles more to the cytoplasm and forms diffuse speckles

Since *Malat1* is known to localize primarily to the nucleus and forms a characteristic speckled pattern (24), we first examined the localization of *Malat1* in ΔSINE cells using single molecule RNA fluorescence *in situ* hybridization (smFISH) (Materials and Methods). *Neat1*, another conserved and abundant lncRNA that also localizes to the nucleus (24), was co-stained with *Malat1* to determine if there were specific changes in the pattern of *Malat1* localization elicited by the SINE deletion. Compared with the wildtype (WT), ΔSINE mutant clones have a significantly higher proportion of cells with cytoplasmic *Malat1* as well as cytoplasmic *Neat1*, although the majority of *Malat1* and *Neat1* speckles are still observed in the nucleus (Figure 1C–E; Supplementary Figure S1A). These data suggest that ΔSINE induced several global cellular changes, possibly including mitotic arrest that resulted in an increase of both cytoplasmic *Malat1* and *Neat1*.

To ensure that the effects observed on cytoplasmic *Malat1* are specific to the SINE deletion, we deleted another region on *Malat1* that does not overlap with the SINE and has previously been implicated in cytoplasmic export of *Malat1* abbreviated as CER (Cytoplasmic Export Region) (Supplementary Figure S1B and C) (15). The CER is 1655-bp in length, which is ∼11-fold larger than the SINE (Supplementary Figure S1B). The CER completely covers fragments J and I reported in Miyagawa et al. (15) (Supplementary Figure S1B) to localize exclusively to the cytoplasm upon overexpression suggesting their potential role in the cytoplasmic export of *Malat1*. Thus, CER deletion (ΔCER) is predicted to promote retention of *Malat1* in the nucleus. Analysis of RNA duplexes generated in Lu *et al.* (14) for RNA structures in living cells reveal that the SINE, a non-conserved element on *Malat1*, is not involved in formation of intramolecular duplexes (e.g. secondary structures) as the relative location of the SINE does not overlap with any regions that form duplexes on *Malat1* (Figure 1F). In contrast, the CER overlaps with many duplex regions (Figure 1F), and hence CER deletion is more likely to disrupt the structure of *Malat1*. As predicted, ΔCER cells displayed a significantly decreased proportion of cells with cytoplasmic *Malat1* but not *Neat1* as compared with the WT (Figure 1E).

Since mitotic cells with nuclear membrane breakdown can cause global cytoplasmic export of nuclear RNAs to the cytoplasm, we further classified cells with cytoplasmic *Malat1* into mitotic and interphase (i.e. non-mitotic) cells based on DAPI signal intensity and texture (Materials and Methods) to determine whether *Malat1* ΔSINE still localizes more to the cytoplasm in non-mitotic cells. Our custom-made scripts effectively distinguished interphase from mitotic cells (Supplementary Figure S1D), which have significantly smaller nuclei than interphase cells (Supplementary Figure S1E). Interphase ΔSINE cells have a significantly higher proportion of cytoplasmic *Malat1* than WT cells, whereas interphase ΔCER cells have a reduced proportion of cytoplasmic *Malat1* (Figure 1G). The difference is not as significant between mitotic ΔSINE, ΔCER and WT cells, although there is a trend of increased cytoplasmic *Malat1* proportions in ΔSINE cells (Figure 1G). Furthermore, the distribution of cytoplasmic *Malat1* of cells in all phases of the cell cycle as a whole is largely contributed by interphase cells as demonstrated by the similarity between the interphase and all-phases cytoplasmic *Malat1* distribution profiles (Figure 1G). This is likely due to a much larger fraction of interphase than mitotic cells. To minimize the effect of the possibility that the script mistakenly classifying mitotic cells as interphase cells, we examined whether interphase ΔSINE cells with much larger nuclei than mitotic WT cells still have more cytoplasmic *Malat1*. Indeed, interphase ΔSINE cells with nuclear areas that are twice the average nuclear area of WT mitotic cells, which are larger than that of mitotic ΔSINE cells (Supplementary Figure S1E), still have a significantly higher cytoplasmic *Malat1* proportion than interphase WT cells (Supplementary Figure S1F). Taken together, these results clearly demonstrate that *Malat1* ΔSINE shuttles more to the cytoplasm in non-mitotic cells.

In addition to localization, we examined changes in the size of *Malat1* speckles since these speckles co-localize with SC35 nuclear speckles (24), which have important roles in coordination of pre-mRNA processing and transcription factors within the nucleus (27). *Malat1* ΔSINE speckles are more diffuse and smaller than WT and *Malat1* ΔCER speckles (Figure 1H and I and Supplementary Figure S1G). In contrast, *Neat1*, which is an essential structural component of paraspeckles in the nucleus (28) had no significant change in speckle size between the mutants and the WT (Figure 1H and I and Supplementary Figure S1G). These data suggests that ΔSINE but not ΔCER causes specific changes to the speckle size of *Malat1* but not *Neat1*. In addition to changes in *Malat1* speckles, *Malat1* expression also increased across all clones of ΔSINE and ΔCER cells (Figure 1J), possibly as a compensation mechanism for the loss of function of *Malat1*. Collectively, these results suggest that the loss of the SINE specifically causes formation of more cytoplasmic and diffuse *Malat1* speckles, which may impact the dynamic stoichiometry of many RBPs known to interact with *Malat1* (29) resulting in subsequent effects on many aspects of transcription and RNA metabolism. In fact, a recent study has demonstrated the importance of *Malat1* in regulating the localization and organization of proteins and RNAs in nuclear speckles (30), implicating a central role of the SINE rather than the CER in dictating such functions of *Malat1*.

### The loss of the SINEB1 in *Malat1* causes apoptosis, DNA damage, ER stress and PKR activation, which corresponds with mitotic defects

**Figure 2.**
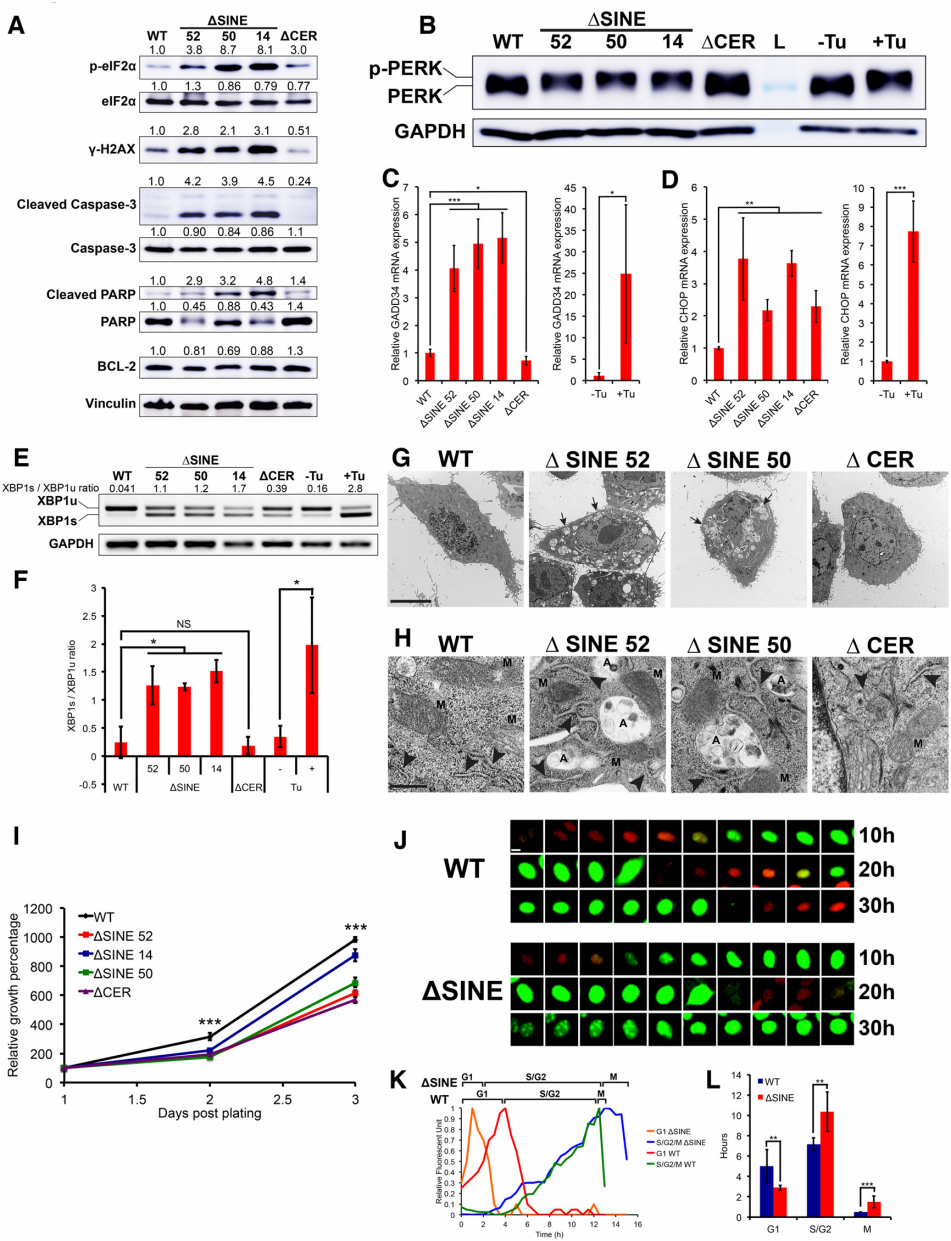
SINE-deleted *Malat1* cells are highly apoptotic with severe ER stress and exhibit a cell cycle delay in S/G2 and M phases. (**A**) ΔSINE cells are highly apoptotic with increased DNA damage and phosphorylation of eIF2α (p-eIF2α). Western blots on lysates from WT, ΔSINE and ΔCER cells. Normalized relative changes in band intensity for individual targets were quantified by ImageJ 1.48v and are shown above each blot. Shown is a representative of three biological replicates. (**B**) ΔSINE cells have increased PERK phosphorylation (p-PERK), a major sensor of ER stress. Western blots on lysates from WT, ΔSINE and ΔCER cells together with Tunicamycin- (Tu) treated cells as positive controls for PERK phosphorylation induction. The upward shift in PERK indicates PERK phosphorylation. Shown is a representative of three biological replicates. L, ladder. (**C** and **D**) ΔSINE cells upregulate GADD34 and CHOP, downstream targets of phosphorylated PERK. Bar graphs comparing relative mRNA levels of GADD34 and CHOP in (D) and (E), respectively. *P < 0.05; **P < 0.01; ***P < 0.001; error bars indicate standard deviation; *n* = 3 biological replicates. (**E**) SINE deletion promotes formation of spliced XBP1 (XBP1s), a downstream target of activated IRE1α, another major sensor of ER stress. Gel electrophoresis images for visualization of XBP1s from XBP1u (unspliced XBP1) bands. Shown is a representative of 3 biological replicates. (**F**) Comparisons of XBP1s/XBP1u ratios between ΔSINE and ΔCER versus WT cells. *P < 0.05; NS, not significant (*P* > 0.05); error bars indicate standard deviation; *n* = 3 biological replicates. (**G**) ΔSINE cells have elevated levels of autophagosomes and cytoplasmic vacuoles. Fixed cells were visualized with transmission electron microscopy (TEM) (scale bar = 10 μm). Black arrows indicate a few examples of autophagosomes. (**H**) ΔSINE cells have swollen ER and condensed mitochondria, a feature of apoptotic cells. TEM images of WT, ΔSINE and ΔCER cells (scale bar = 500 nm). Arrow heads indicate ER regions. M, mitochondria; A, autophagosome. (**I**) ΔSINE cells exhibit decreased growth compared to WT cells. Growth curves of all cell types, measured by crystal violet assay. ***P < 0.001; error bars indicate standard deviation; *n* = 3 biological replicates. (**J**) ΔSINE-cells have shortened G1 and prolonged S/G2/M. Live-cell images of Fucci-expressing cells. Red (RFP) indicates G1 cells; green (GFP) indicates S/G2/M cells. Shown is a representative of 20 cells for each group. (**K**) Both S/G2 and mitosis are delayed in SINE-deleted cells. Relative fluorescent intensity of RFP (red and orange) and GFP (green and blue) in Fucci-tagged cells during the time course of one cell cycle (scale bar = 10 μm). Shown is a representative of 20 cells for each group. (**L**) Quantification of the number of hours the cell stays in each phase of cell cycle. Cell cycle was tracked using Fucci reporters. **P < 0.01; ***P < 0.001; error bars indicate standard deviation; *n* = 20 individual cells for each group.

Since SINE and Alu have been implicated in regulation of translation (3,26), we performed polysome analysis to investigate translational changes potentially induced by *Malat1* ΔSINE. Unexpectedly, we observed a reduction in global translation in ΔSINE cells, in which a relatively smaller fraction of RNA was bound to polysomes that represent actively translating ribosomes (Supplementary Figure S2A). We confirmed a relatively large increase in phosphorylation of eIF2α, a central regulator of translation initiation whose phosphorylation inhibits global protein synthesis (31), in both ΔSINE and ΔCER cells (Figure 2A). Levels of γ-H2AX, cleaved Caspase-3 and cleaved PARP markedly increased in ΔSINE but not ΔCER cells with a concomitant reduction in total Caspase-3 and PARP levels (Figure 2A), suggesting that *Malat1* ΔSINE selectively induced DNA damage and apoptosis. BCL-2, an anti-apoptotic protein localized to the outer membrane of mitochondria (32), also decreased in protein expression (Figure 2A), suggesting the involvement of the canonical mitochondrial cell death pathway. In contrast, ΔCER, which promoted *Malat1*’s nuclear retention, seemed to exert protective effects against DNA damage and apoptosis by decreasing the levels of γ-H2AX and cleaved Caspase-3 and increasing the expression of BCL-2 (Figure 2A).

eIF2α is an essential member of the integrated stress response (ISR) pathway and is phosphorylated by kinases that sense distinct environmental and physiological stresses (33). We found that PERK and PKR, direct eIF2α kinases and sensors of the endoplasmic reticulum (ER) stress and double-stranded RNAs (dsRNAs), respectively (33), had increased phosphorylation in ΔSINE but not ΔCER cells (Figure 2B and Supplementary Figure S2B). ΔSINE cells also exhibited increased dsRNA levels (Supplementary Figure S2C) and upregulation of GADD34 and CHOP (Figure 2C and D), downstream targets of the PERK-eIF2α axis induced as a part of the ISR (33). The increased CHOP expression in ΔCER cells is possibly due to the elevated eIF2α phosphorylation (Figure 2D) induced by cellular events other than ER stress and dsRNAs. In addition to PERK phosphorylation, we confirmed activation of IRE1α, a different arm of UPR activation, through detection of increased XBP1 pre-mRNA splicing (Figure 2E and F), suggesting global UPR activation by *Malat1* ΔSINE. Electron microscopy revealed that ΔSINE cells had many autophagosomes and cytoplasmic vacuoles (Figure 2G), implicating the activation of the autophagy pathway possibly as a survival response to ER stress (34). The cells also had enlarged ER, a well-known feature of cells with ER stress (35), and condensed mitochondria with loss/unfolding of cristae structure (Figure 2G), a feature of pre-apoptotic cells that has been shown to facilitate cytochrome c release from fragmented mitochondria during apoptosis (36). Taken together, these results suggest that the SINEB1 of *Malat1* is essential for cell survival, genomic integrity, proper protein folding and minimizing PKR activation.

The multiple stresses induced by *Malat1* ΔSINE are accompanied by slower growth (Figure 2I) and cell cycle defects, including shortened G1 (∼3 h versus ∼5 h) and prolonged S/G2 (∼10 h versus ∼8 h) and M phases (∼1 h versus ∼0.5 h) as compared with WT cells (Figure 2J–L, and Supplementary Figure S2D and E). The ΔSINE cells also have many micronuclei fragmented from the main nuclei (Supplementary Figure S2F and G) and abnormally aggregated phospho-Histone H3 (p-H3) staining (Supplementary Figure S2G and I). Live-cell imaging with Fucci reporters confirmed that a high frequency of ΔSINE cells have defects in cytokinesis and cell segregation at the end of mitosis resulting in mitotic catastrophe (Supplementary Video S1–S3 and Figure S2J). The latter is known to be induced by DNA damage due to PARP depletion (37) caused by caspase 3 activation (Figure 2A).

### *Malat1* ΔSINE induces apoptosis by promoting cytoplasmic mis-localization of TDP-43 and formation of cytotoxic TDP-43 inclusions, which are further amplified by caspase-3-induced formation of the aggregate-prone C-terminal 35 kDa TDP-43 fragment

**Figure 3.**
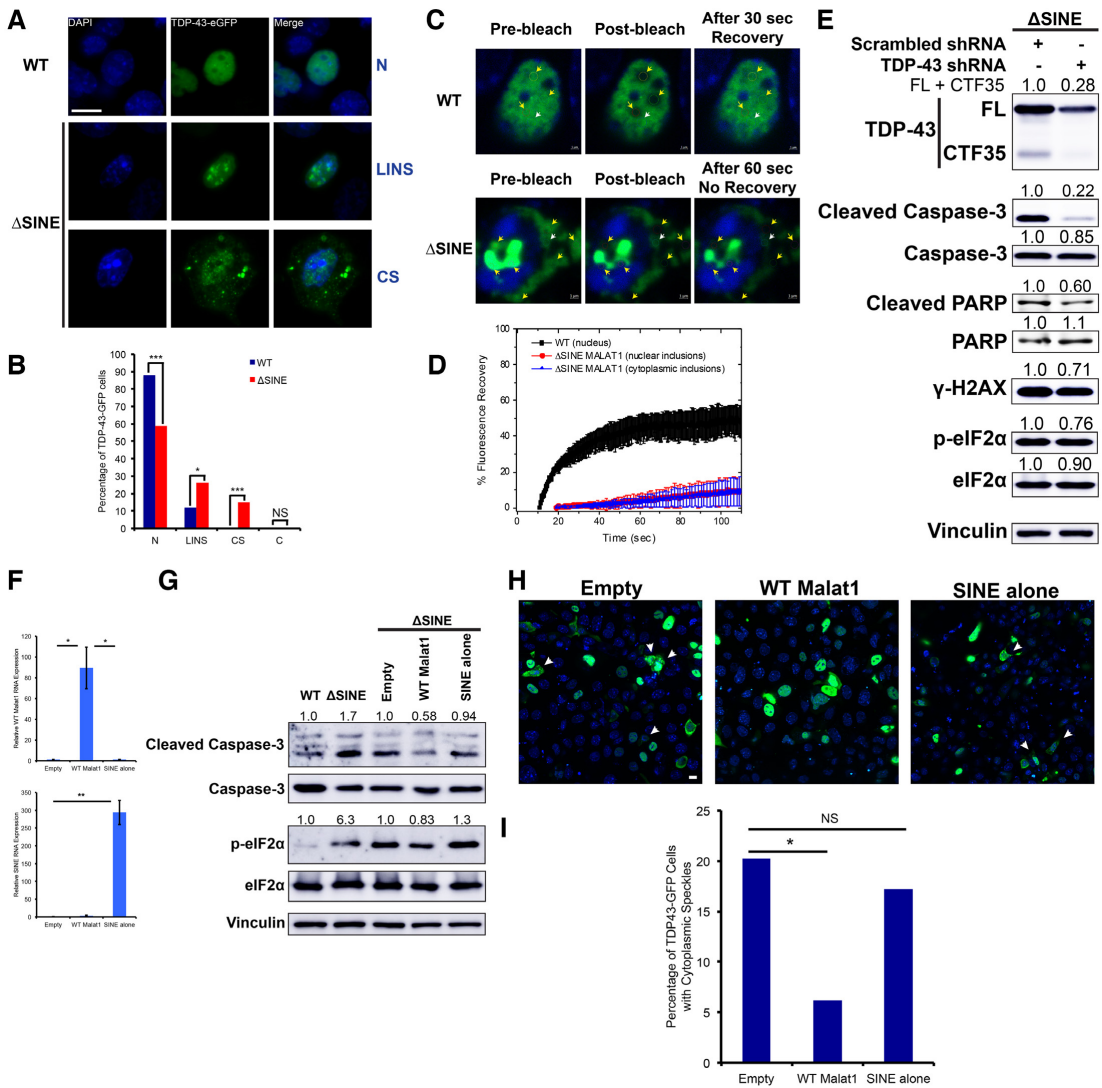
*Malat1* ΔSINE induces apoptosis through induction of TDP-43 aggregation. (**A**) Exogenously expressed full-length TDP-43 forms nuclear and cytoplasmic speckles with irregular shapes in ΔSINE cells, indicative of aggregate formation instead of liquid–liquid phase separation, which has a round shape. Live images of cells transfected with the full-length TDP-43-GFP construct (scale bar = 10 μm). N, cells with only nuclear TDP-43 but no speckle or just a few tiny round ones; LINS, cells with large irregular shaped nuclear TDP-43 speckles; CS, cells with cytoplasmic TDP-43 speckles. Note that cells with these speckles appear unhealthy, possibly undergoing apoptosis. (**B**) Cells with large irregular shaped nuclear and cytoplasmic TDP-43 speckles are significantly more prevalent in ΔSINE cells, and cells with only nuclear TDP-43 are not as common in ΔSINE cells compared with WT cells, suggesting an increase in cytoplasmic localization of TDP-43. Quantification of cells with different TDP-43-GFP localization patterns. N, cells with only nuclear TDP-43 but no speckle or just a few tiny round ones; LINS, cells with large irregular shaped nuclear TDP-43 speckles; CS, cells with cytoplasmic TDP-43 speckles; C, cells with only cytoplasmic TDP-43 but no detectable speckle. *Pearson's* chi-squared test was used for comparison. **P < 0.01; ***P < 0.001; NS, not significant (*P* > 0.05); *n* ≈ 155 cells for each group. (**C**) *Malat1* ΔSINE dramatically promotes the formation of insoluble aggregates of TDP-43,. Images of FRAP analysis for TDP-43-GFP in WT and ΔSINE cells (scale bar = 1 μm). Yellow arrows indicate photobleached areas. White arrows indicate areas without photobleaching as references. Note that both nuclear and cytoplasmic TDP-43-GFP in ΔSINE cells could not recover GFP signal even 60 s post bleaching, whereas 30 s was sufficient for GFP to fully recover in WT cells. (**D**) Time-lapse quantification of TDP-43-GFP fluorescence recovery after photobleaching, demonstrating a drastic reduction in the liquid-like property of TDP-43 in ΔSINE cells. (**E**) TDP-43 depletion rescues apoptosis in ΔSINE cells, suggesting that TDP-43 aggregation is the cause of cellular toxicity. Western blots on lysates from ΔSINE cells stably expressing scrambled shRNA or TDP-43 shRNA. Shown is a representative of 3 biological replicates. (**F**) Stable overexpression of WT *Malat1* and the SINE alone in ΔSINE cells. Overexpression relative to the empty vector was confirmed with qPCR. *P < 0.05; **P < 0.01; error bars indicate standard deviation; *n* = 3 biological replicates. (**G**) WT *Malat1* overexpression rescues apoptosis and reduces eIF2α phosphorylation in cells with the mutant *Malat1* ΔSINE, suggesting that SINE deletion causes a loss of function of *Malat1*. WT and ΔSINE cells serve as reference controls. Shown is a representative of three biological replicates. (**H**) Overexpression of WT *Malat1* reduces formation of cytotoxic TDP-43 cytoplasmic speckles in ΔSINE cells. Live images of cells transfected with the full-length TDP-43-GFP construct (scale bar = 10 μm). (**I**) Quantification of the percentage of TDP-43-GFP cells with cytoplasmic speckles in ΔSINE cells with overexpression of WT *Malat1* or the SINE alone. *Pearson's* chi-squared test was used for comparison. *P < 0.05; NS, not significant (*P* > 0.05); *n* ≈ 100 cells for each group.

TDP-43, an abundant and predominantly nuclear protein with central roles in RNA metabolism is capable of forming insoluble cytotoxic protein aggregates (38), and has been shown to exhibit increased binding to *Malat1* in neurodegenerative brain tissues with cytoplasmic TDP-43 inclusions (39). Moreover, TDP-43 depletion also results in an increased fraction of cells in S and G2/M and induction of apoptosis (40,41), similar to phenotypes caused by *Malat1* ΔSINE. Therefore, we investigated whether the *Malat1* ΔSINE might promote formation of cytoplasmic TDP-43 inclusions and depletion of biologically active TDP-43. Exogenously expressed full length TDP-43-eGFP localizes more to the cytoplasm of ΔSINE as compared with WT cells as demonstrated by a significant reduction in the number of cells with nuclear-restricted TDP-43 and a significant increase in the number of cells with TDP-43 cytoplasmic speckles (Figure 3A and B). Cytoplasmic TDP-43 has been shown to induce mitochondrial dysfunction and fragmentation, which precede apoptosis and caspase-3 activation, by blocking mitochondrial RNAs’ translation (42), consistent with the elevated level of cleaved caspase-3 in ΔSINE cells. More large irregular shaped TDP-43 speckles, indicative of cytotoxic inclusions, are found in both the nucleus and cytoplasm of ΔSINE cells (Figure 3A and B and Supplementary Figure S3A). To confirm the formation of TDP-43 inclusions in *Malat1* ΔSINE cells, Fluorescence Recovery After Photobleaching (FRAP) was performed on the eGFP signal of exogenously expressed TDP-43. A dramatic reduction was observed in the ability of bleached TDP-43-eGFP in both the nucleus and cytoplasm of *Malat1* ΔSINE cells to recover (Figure 3C and D), indicating that TDP-43 in ΔSINE cells exhibit insoluble condensate properties compared with the more biologically active soluble properties of TDP-43 in WT cells. Collectively, these results suggest that *Malat1* ΔSINE promotes the formation of cytotoxic insoluble TDP-43 inclusions in both the nucleus and cytoplasm.

We also observed a reduction in the level of endogenous full length TDP-43 (FL) in ΔSINE cells and an increase in the level of C-terminal 35 kDa TDP-43 fragment (CTF35) (Supplementary Figure S3B), which has lost the nuclear localization signal and is prone to forming aggregates (43–45). This marked increase in CTF35 is due to the elevation of cleaved caspase-3 in ΔSINE cells since caspase-3 can cleave TDP-43 into CTF-35. Furthermore, caspase inhibition reduced TDP-43 CTF35 levels as well as cleaved PARP, a caspase-3 substrate (Supplementary Figure S3C). The reduction in TDP-43 FL implicates a loss of function of WT TDP-43 (Supplementary Figure S3B), which also may explain the cell cycle defects observed in ΔSINE cells since TDP-43 depletion has been demonstrated to cause an increase in S and G2/M cells (40). Nuclear-cytoplasmic fractionation further confirmed increased levels of both TDP-43 FL and CTF35 in the cytoplasm of *Malat1* ΔSINE cells (Supplementary Figure S3D), consistent with cytoplasmic inclusions observed in ΔSINE cells with exogenously expressed TDP-43-eGFP (Figure 3A-D and Supplementary Figure S3A). It is worth noting that the increase in cytoplasmic TDP-43 FL shown in Supplementary Figure S3D is likely underestimated since only soluble TDP-43 was measured but a large increase in the number of cells with cytoplasmic insoluble TDP-43 inclusions was observed (Figure 3B). Taken together, these data suggest that involvement of TDP-43 CTF35 in forming cytotoxic aggregates in *Malat1* ΔSINE cells and that cell cycle defects observed in these cells are potentially due to depletion of WT TDP-43 FL.

If TDP-43 forms cytotoxic inclusions, its depletion should rescue apoptosis in *Malat1* ΔSINE cells. Indeed, TDP-43 knockdown normalized the levels of cleaved Caspase-3 and cleaved PARP in ΔSINE cells, but minimal changes were detected in WT cells (Figure 3E and Supplementary Figure S3E). Depletion of TDP-43 slightly decreased γ-H2AX and p-eIF2α levels in ΔSINE cells, but increased γ-H2AX levels in WT cells (Figure 3E and Supplementary Figure S3E), suggesting that a loss of function of TDP-43 promoted DNA damage under normal conditions and hence partly negated the rescue effect of TDP-43 knockdown on DNA damage induced by *Malat1* ΔSINE. This is consistent with known roles of TDP-43 in the prevention or repair of R loop-associated DNA damage (46). Furthermore, overexpression of WT *Malat1* in ΔSINE cells reduced the levels of cleaved Caspase-3 and p-eIF2α (Figure 3F and G) as well as the proportion of cells with cytoplasmic TDP-43 inclusions (Figure 3H and I), implicating that observed phenotypes are due to a loss of function of *Malat1*. In contrast, overexpression of the SINE alone had no effect (Figure 3F-I). These results together suggest that *Malat1* ΔSINE induces apoptosis primarily by inducing formation of cytotoxic TDP-43 inclusions.

### TDP-43-inclusion-induced apoptosis in *Malat1* ΔSINE cells is partially dependent on ER stress and PKR activation

Since apoptosis is a possible downstream effect of stress sensor activation, we asked whether TDP-43 aggregates induce apoptosis through PERK and PKR activation, both of which are known to be induced by defects in TDP-43 function (47,48). Depletion of PERK or PKR alone has minimal effect on cleaved caspase-3 levels but knockdown of both PERK and PKR partially reduced cleaved caspase-3 levels (Supplementary Figure S4A–C), suggesting that ΔSINE *Malat1* drives TDP-43-inclusion-induced apoptosis partly through PERK and PKR activation. Levels of eIF2α phosphorylation and γ-H2AX (Supplementary Figure S4C) as well as GADD34 and CHOP expression (Supplementary Figure S4D and E) were not affected by PERK and PKR depletion. These data suggest that PERK and PKR activation are not the primary routes taken by TDP-43 inclusions for apoptosis induction.

Furthermore, knockdown of TDP-43 was not sufficient to rescue ER stress in ΔSINE cells as shown by the absence of significant changes in GADD34 and CHOP expression as well as the XBP1s/XBP1u ratio in ΔSINE cells (Supplementary Figure S4F–J). PERK and PKR knockdown alone or in combination were also unable to reduce the level of TDP-43 CTF35 in ΔSINE cells (Supplementary Figure S4K), suggesting that the stress sensor activation and TDP-43-inclusion-induced cell death are two independent events caused by *Malat1* ΔSINE.

### *Malat1* ΔSINE has increased binding to TDP-43 and reprograms TDP-43 binding to mitotic and nuclear-cytoplasmic transport regulators and repetitive element transcripts

**Figure 4.**
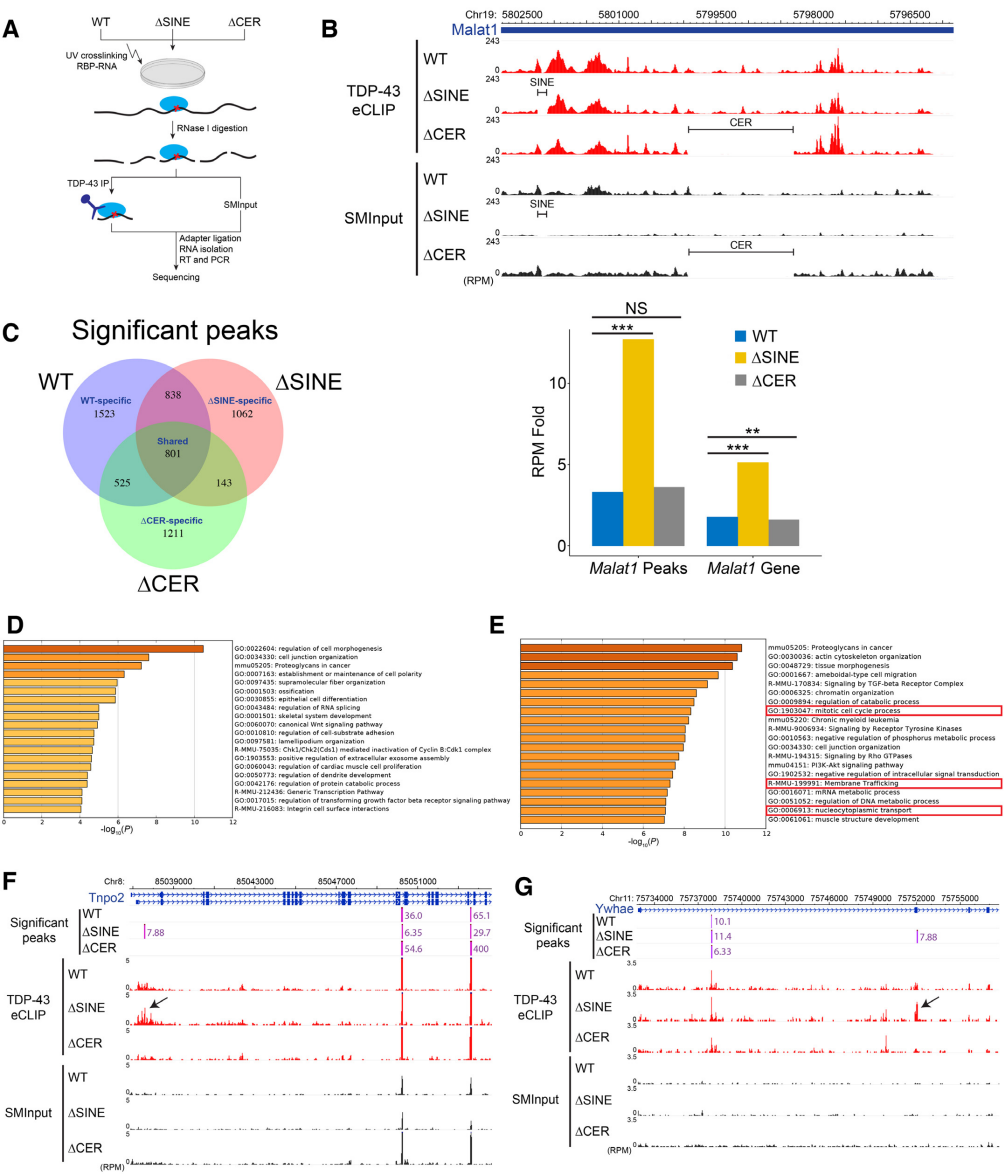
*Malat1* ΔSINE binds stronger to TDP-43 and reprograms TDP-43′s binding on mRNAs of genes involved in mitotic regulation and membrane trafficking. (**A**) Schematic overview of TDP-43 eCLIP-seq on WT, ΔSINE and ΔCER cells. SMInput, size-matched input; RBP, RNA-binding protein; RT, reverse transcription. (**B**) Although the binding pattern of TDP-43 on *Malat1* does not change in SINE-deleted cells, *Malat1* ΔSINE binds significantly stronger to TDP-43 than WT and ΔCER *Malat1*. Top: TDP-43 eCLIP read density tracks along *Malat1* in reads per million (RPM) with paired SMInput for each cell type. Note that SINE and CER regions have almost no reads in ΔSINE and ΔCER cells, respectively and that TDP-43 does not bind to the SINE region. Bottom: Bars comparing enriched RPM folds of TDP-43 eCLIP reads across all *Malat1* significant peak regions (*Malat1* Peaks) or the full *Malat1* gene body (*Malat1* Gene). RPM fold was quantified by dividing normalized RPMs of TDP-43 IP over that of SMInput. **P < 0.01; ***P < 0.001; NS, not significant (*P* > 0.05). *P* values were calculated using *Pearson's* chi-squared test. (**C**) Both ΔSINE and ΔCER cells have TDP-43 binding sites that are distinct from WT cells. Venn diagram demonstrating significant TDP-43 eCLIP peaks. Significant peaks are >2 fold-enriched, *P* < 0.01 above SMInput. (**D**) TDP-43 predominantly binds to genes involved in cell morphogenesis and junction organization. Cellular functions and pathways enriched in genes with TDP-43 binding sites that are shared between WT, ΔSINE and ΔCER cells using Metascape. (**E**) Many TDP-43 binding sites unique for SINE-deleted cells are found in genes involved in mitotic regulation and membrane trafficking. Cellular functions and pathways enriched in genes with ΔSINE-specific TDP-43 binding using Metascape. (**F** and **G**) SINE deletion promotes binding of TDP-43 to an exon of *Ywhae*, a mitotic regulator, and an intron of *Tnpo2*, a nuclear-cytoplasmic transport regulator, respectively. Shown are the *Ywhae* and *Tnpo2* read densities. Average enriched folds above SMInput are shown adjacent to significant peaks (*P* < 0.01). Arrow indicates the region with increased TDP-43 binding.

To investigate whether SINE deletion causes alterations in TDP-43 binding to *Malat1* and other transcripts, we performed eCLIP-seq to capture and identify genome-wide TDP-43 binding sites on RNAs of WT, ΔSINE and ΔCER cells (Figure 4A and Supplementary Figure S5A). The TDP-43 binding pattern on *Malat1* is largely unchanged in ΔSINE and ΔCER cells versus WT since both the SINE and the CER do not overlap with *Malat1* regions that have a high density of TDP-43 binding sites (Figure 4B). However, TDP-43 binds significantly stronger to *Malat1* ΔSINE than *Malat1* ΔCER and WT *Malat1* as demonstrated by a marked increase in eCLIP-seq reads per million (RPM) fold normalized by size-matched input (SMInput) (Figure 4B), suggesting that deletion of the SINEB1 in *Malat1* promotes sponging of TDP-43 to *Malat1*. In the global transcriptome, both ΔSINE and ΔCER cells harbor TDP-43 binding programs that are distinct from WT, in addition to those shared by all three genotypes (Figure 4C). Interestingly, unlike genes with shared TDP-43 binding, many genes with SINE-specific TDP-43 binding sites are involved in regulation of mitosis, cell cycle processes, membrane trafficking and nuclear-cytoplasmic transport (Figure 4D and E and Table 1). These cellular functions are not enriched in genes with WT- and CER-specific TDP-43 binding sites (Supplementary Figure S5B and S5C). TDP-43 showed enhanced binding to both exonic and intronic regions of target transcripts (Figure 4F and G, and Supplementary Figure S5D and E). These alterations in TDP-43 binding previously have been demonstrated to cause RNA mis-splicing in neurodegenerative diseases (49). Furthermore, we found that while in all genotypes TDP-43 still binds to UG-rich motifs that are the well-documented binding sites of TDP-43 (49), TDP-43 binding sites in ΔSINE cells are significantly enriched with an additional GA-rich motif, which is not found in WT and ΔCER cells (Supplementary Figure S5F). TDP-43 has previously been reported to specify HPL-2 binding to DNA regions with the GA-rich motif in *Caenorhabditis elegans* (50), suggesting that *Malat1* ΔSINE either shifted TDP-43 binding from a UG-rich to a GA-rich motif or promoted TDP-43 binding to more GA-rich regions.

**Table 1.** Genes with SINE-specific peaks involved in cell cycle and cellular transport regulation (>2 fold-enriched, *P* < 0.01 above SMInput)

Function	Gene and description
Mitosis, cell cycle	Incenp: inner centromere protein
	Spc24: SPC24, NDC80 kinetochore complex component, homolog (*S. cerevisiae*)
	Ahctf1: AT hook containing transcription factor 1
	Nek2: NIMA (never in mitosis gene a)-related expressed kinase 2
	Ywhag: tyrosine 3-monooxygenase/tryptophan 5-monooxygenase activation protein, gamma polypeptide
	Smc1a: structural maintenance of chromosomes 1A
	Rangap1: RAN GTPase activating protein 1
	Mcm3: minichromosome maintenance deficient 3 (*S. cerevisiae*)
	Nup93: nucleoporin 93
	Nup188: nucleoporin 188
	Cenpc1: centromere protein C1
	Tuba1a: tubulin, alpha 1A
	Ppp2r2d: protein phosphatase 2, regulatory subunit B, delta isoform
	Cdkn1a: cyclin-dependent kinase inhibitor 1A (P21)
	Dyrk1a: dual-specificity tyrosine-(Y)-phosphorylation regulated kinase 1a
	Rcc2: regulator of chromosome condensation 2
	Cdc25b: cell division cycle 25B
	Ppp2r2a: protein phosphatase 2 (formerly 2A), regulatory subunit B (PR 52), alpha isoform
	Mcm5: minichromosome maintenance deficient 5, cell division cycle 46 (*S. cerevisiae*)
	Ywhae: tyrosine 3-monooxygenase/tryptophan 5-monooxygenase activation protein, epsilon polypeptide
	E2f3: E2F transcription factor 3
	Cenpi: centromere protein I
	Rfc3: replication factor C (activator 1) 3
	Kif23: kinesin family member 23
	Ccnd1: cyclin D1
	Csnk1d: casein kinase 1, delta
	Taok1: TAO kinase 1
	Uba52: ubiquitin A-52 residue ribosomal protein fusion product 1
Membrane tracking,	Tnpo2: transportin 2 (importin 3, karyopherin beta 2b)
nuclear-cytoplasmic	Lrp12: low density lipoprotein-related protein 12
transport	Ddx58: DEAD (Asp-Glu-Ala-Asp) box polypeptide 58
	Snx9: sorting nexin 9
	Mon2: MON2 homolog (yeast)
	Sft2d3: SFT2 domain containing 3
	Atp2b1: ATPase, Ca^2+^ transporting, plasma membrane 1
	Atad1: ATPase family, AAA domain containing 1
	Micall2: MICAL-like 2
	Dynll2: dynein light chain LC8-type 2
	Rab14: RAB14, member RAS oncogene family
	Scfd1: Sec1 family domain containing 1
	Osbpl6: oxysterol binding protein-like 6
	Slc25a33: solute carrier family 25, member 33
	Ipo5: importin 5
	Ubr5: ubiquitin protein ligase E3 component n-recognin 5
	Pard3: par-3 (partitioning defective 3) homolog (*C. elegans*)
	Slc26a11: solute carrier family 26, member 11
	Naif1: nuclear apoptosis inducing factor 1
	Osbpl3: oxysterol binding protein-like 3
	Nup93: nucleoporin 93
	Strada: STE20-related kinase adaptor alpha
	Uaca: uveal autoantigen with coiled-coil domains and ankyrin repeats
	Golim4: golgi integral membrane protein 4
	Calm2: calmodulin 2
	Kcnh5: potassium voltage-gated channel, subfamily H (eag-related), member 5
	Cfl1: cofilin 1, non-muscle
	Smurf1: SMAD specific E3 ubiquitin protein ligase 1
	Ipo11: importin 11
	Slc10a7: solute carrier family 10 (sodium/bile acid cotransporter family), member 7
	Akt1: thymoma viral proto-oncogene 1
	Atp2b2: ATPase, Ca^2+^ transporting, plasma membrane 2
	Slc7a1: solute carrier family 7 (cationic amino acid transporter, y+ system), member 1
	Bcl2l1: BCL2-like 1
	Bmp4: bone morphogenetic protein 4
	Ldlrad3: low density lipoprotein receptor class A domain containing 3
	Hdlbp: high density lipoprotein (HDL) binding protein
	Ccnd1: cyclin D1
	Cse1l: chromosome segregation 1-like (*S. cerevisiae*)
	Slc18a1: solute carrier family 18 (vesicular monoamine), member 1
	Cdkn1a: cyclin-dependent kinase inhibitor 1A (P21)
	Chuk: conserved helix-loop-helix ubiquitous kinase
	Pex10: peroxisomal biogenesis factor 10
	Cp: ceruloplasmin
	Csf3: colony stimulating factor 3 (granulocyte)
	Wasf2: WAS protein family, member 2
	Egfr: epidermal growth factor receptor
	Derl1: Der1-like domain family, member 1
	Stx2: syntaxin 2
	Epn2: epsin 2
	F2rl1: coagulation factor II (thrombin) receptor-like 1
	Fkbp1a: FK506 binding protein 1a
	Aktip: thymoma viral proto-oncogene 1 interacting protein
	Fzd1: frizzled homolog 1 (Drosophila)
	Grb2: growth factor receptor bound protein 2
	Grin2a: glutamate receptor, ionotropic, NMDA2A (epsilon 1)
	Hk2: hexokinase 2
	Agfg1: ArfGAP with FG repeats 1
	Hspa4: heat shock protein 4
	Hspa9: heat shock protein 9
	Rrbp1: ribosome binding protein 1
	Kcnk1: potassium channel, subfamily K, member 1
	Kif5b: kinesin family member 5B
	Kif5c: kinesin family member 5C
	Uhmk1: U2AF homology motif (UHM) kinase 1
	Psen1: presenilin 1
	Ldlr: low density lipoprotein receptor
	Lif: leukemia inhibitory factor
	Anxa1: annexin A1
	Lrp5: low density lipoprotein receptor-related protein 5
	Smad2: SMAD family member 2
	Smad3: SMAD family member 3
	Smad4: SMAD family member 4
	Slc3a2: solute carrier family 3 (activators of dibasic and neutral amino acid transport), member 2
	Laptm4a: lysosomal-associated protein transmembrane 4A
	Myo10: myosin X
	Nnat: neuronatin
	Mybbp1a: MYB binding protein (P160) 1a
	Pam: peptidylglycine alpha-amidating monooxygenase
	Prkcd: protein kinase C, delta
	Sec22c: SEC22 vesicle trafficking protein homolog C (*S. cerevisiae*)
	Ppard: peroxisome proliferator activator receptor delta
	Tnk2: tyrosine kinase, non-receptor, 2
	Cnksr3: Cnksr family member 3
	Nus1: nuclear undecaprenyl pyrophosphate synthase 1 homolog (*S. cerevisiae*)
	Ptpn11: protein tyrosine phosphatase, non-receptor type 11
	Ptpn14: protein tyrosine phosphatase, non-receptor type 14
	Rab7: RAB7, member RAS oncogene family
	Rac1: RAS-related C3 botulinum substrate 1
	Rangap1: RAN GTPase activating protein 1
	Trappc10: trafficking protein particle complex 10
	Eea1: early endosome antigen 1
	Agap1: ArfGAP with GTPase domain, ankyrin repeat and PH domain 1
	Ncor1: nuclear receptor co-repressor 1
	Itsn2: intersectin 2
	Vps4b: vacuolar protein sorting 4b (yeast)
	Slc7a5: solute carrier family 7 (cationic amino acid transporter, y+ system), member 5
	Tmc6: transmembrane channel-like gene family 6
	Tgfbr2: transforming growth factor, beta receptor II
	Thbs1: thrombospondin 1
	Tnks: tankyrase, TRF1-interacting ankyrin-related ADP-ribose polymerase
	Tsg101: tumor susceptibility gene 101
	Vegfa: vascular endothelial growth factor A
	Ywhae: tyrosine 3-monooxygenase/tryptophan 5-monooxygenase activation protein, epsilon polypeptide
	Ywhag: tyrosine 3-monooxygenase/tryptophan 5-monooxygenase activation protein, gamma polypeptide
	Ywhaz: tyrosine 3-monooxygenase/tryptophan 5-monooxygenase activation protein, zeta polypeptide
	Litaf: LPS-induced TN factor
	Sufu: suppressor of fused homolog (Drosophila)
	Xpo4: exportin 4
	Chd7: chromodomain helicase DNA binding protein 7
	Slc3a1: solute carrier family 3, member 1
	Tcirg1: T cell, immune regulator 1, ATPase, H+ transporting, lysosomal V0 protein A3
	Btbd9: BTB (POZ) domain containing 9
	Atp5o: ATP synthase, H+ transporting, mitochondrial F1 complex, O subunit
	Nipa2: non imprinted in Prader-Willi/Angelman syndrome 2 homolog (human)
	Casc3: cancer susceptibility candidate 3
	Flna: filamin, alpha
	Slc10a6: solute carrier family 10 (sodium/bile acid cotransporter family), member 6
	Ehd2: EH-domain containing 2
	Atf4: activating transcription factor 4
	Ahctf1: AT hook containing transcription factor 1
	Prickle1: prickle homolog 1 (Drosophila)
	Slc2a6: solute carrier family 2 (facilitated glucose transporter), member 6
	Nup188: nucleoporin 188
	Tlk1: tousled-like kinase 1
	Syt6: synaptotagmin VI
	Stx16: syntaxin 16
	Xpo7: exportin 7
	Xpot: exportin, tRNA (nuclear export receptor for tRNAs)

We also observed a global increase in TDP-43 binding to repetitive elements including satellites, SINEs and ERV endogenous retrovirus TEs in ΔSINE cells relative to WT and ΔCER cells (Supplementary Figure S5G and H). Interestingly, TDP-43 binding to L1 decreased in both ΔSINE and ΔCER cells (Supplementary Figure S5H), implicating a potential connection between *Malat1* as a whole, L1 and TDP-43. In fact, TDP-43 loss has been associated with increased L1 retrotransposition (51). The increase in binding of TDP-43 to other repetitive elements specifically in ΔSINE cells may be due to TDP-43 aggregation, increased binding to *Malat1* and altered interaction with other RBPs that are also influenced by *Malat1* ΔSINE.

Taken together, our data suggest that *Malat1* ΔSINE exhibits increased binding to TDP-43 and likely hijacks TDP-43 to the cytoplasm through *Malat1*’s cytoplasmic mis-localiztion, promoting TDP-43 aggregation. Furthermore, *Malat1* ΔSINE reprograms the binding of TDP-43 to cellular RNAs including repetitive element transcripts and enhances TDP-43 binding to mRNAs encoding mitotic and nuclear-cytoplasmic transport regulators, potentially impairing their processing and function.

### The SINEB1 of *Malat1* is enriched in RNAs with a relatively high nuclear to cytoplasmic copy number ratio and is required for proper *Malat1* binding to HNRNPK, a RBP known to drive nuclear retention, potentially through direct interactions between the SINEB1 and KHDRBS1 or TR2A RBPs

**Figure 5.**
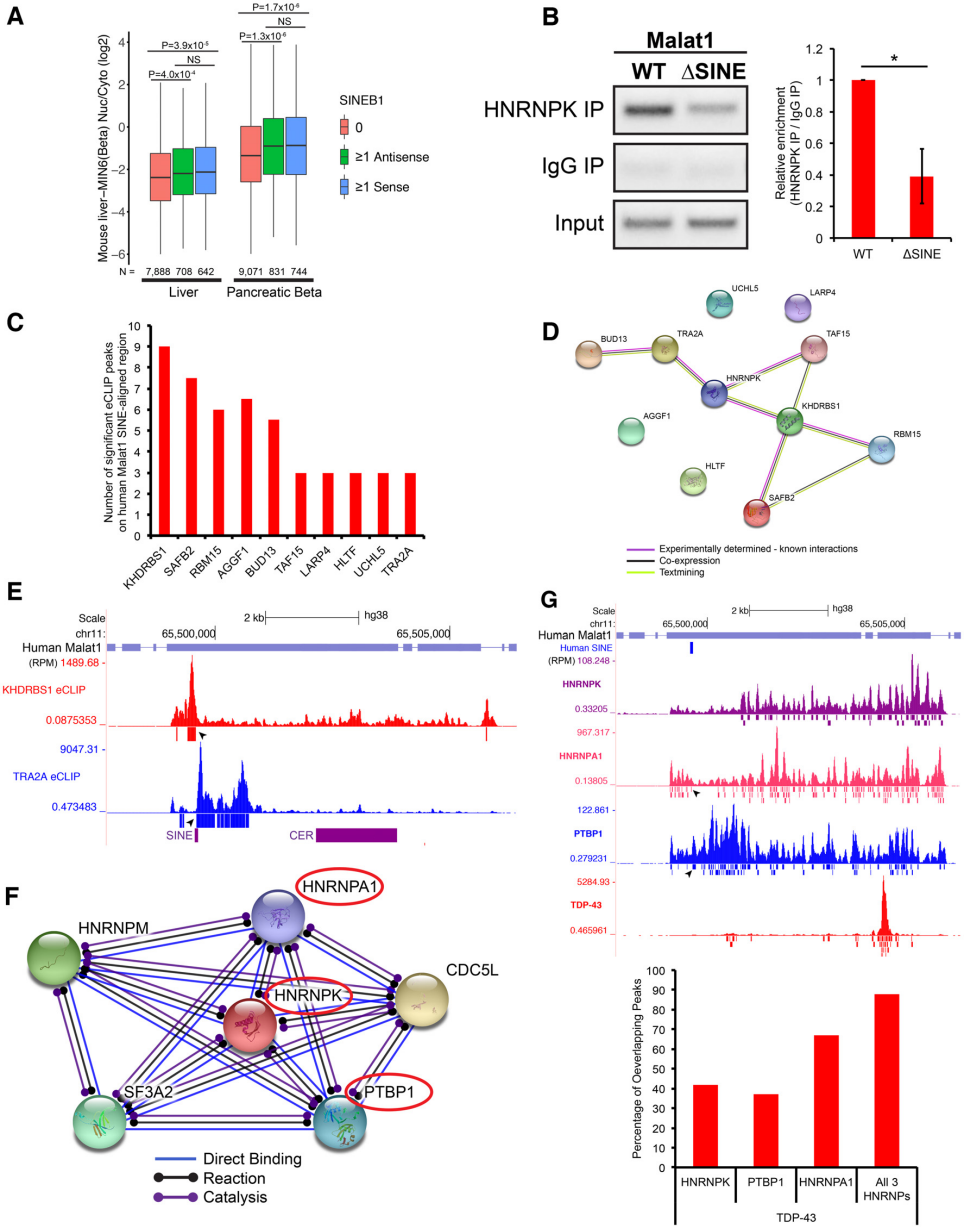
The SINEB1 in *Malat1* is enriched in RNAs with high ratios of nuclear to cytoplasmic copy numbers, and *Malat1* without the SINE has decreased binding to HNRNPK, which likely binds *Malat1* as RBP complexes. (**A**) Copies of the *Malat1* SINEB1, either the sense or antisense strand, are enriched in mRNAs with high nuclear to cytoplasmic copy number ratios. A boxplot comparing the ratio of nuclear to cytoplasmic copy numbers of mRNAs without or with at least one sense or antisense SINEB1 in mouse liver and pancreatic beta cells. The copy number values for the mRNAs in the nucleus and the cytoplasm of the two cell types were determined in Halpern *et al.* (12). NS, not significant (*P* > 0.05). (**B**) *Malat1* ΔSINE has decreased binding to HNRNPK. Left: Gel electrophoresis images of *Malat1* RNA immunoprecipitation (RIP) assay with HNRNPK antibody on lysates from WT and ΔSINE cells. Right: Comparison of *Malat1*’s enrichment in HNRPK IP over IgG IP in WT and ΔSINE cells. Normalized *Malat1* RIP band intensity from gel electrophoresis images was quantified by ImageJ 1.48v. Shown is a representative of three biological replicates. (**C**) A number of RBPs bind to the SINEB1-aligned region on human *Malat1*. Bars indicate eCLIP peak numbers in the top 10 RBPs with the highest number of significant eCLIP peaks on the human SINEB1-aligned region. All significant eCLIP peaks for 120 RBPs in K562 cells were extracted from the ENCODE database and then overlapped with the human SINEB1-aligned region. Note that HNRNPK does not have any eCLIP peaks that overlap the human SINEB1-aligned region. (**D**) Among the top 10 human-SINEB1-bound RBPs, KHDRBS1, TRA2A and TAF15 have been experimentally determined to interact with HNRNPK. Protein-protein interaction network analysis of the top 10 human-SINEB1-bound RBPs together with HNRNPK using STRING database. (**E**) KHDRBS1 and TRA2A’s major binding sites overlap with the SINE but not the CER region on human *Malat1*. KHDRBS1 and TRA2A eCLIP read density tracks along human *Malat1*. Boxes underneath each track highlight the most significant peaks. Black arrow heads indicate peaks that overlap with the human SINE-aligned region. (**F**) Other than interacting with KHDRBS1 and TRA2A, HNRNPK is known to bind other RBPs such as HNRNPA1 and PTBP1, both of which also have binding sites in the SINE region as shown in Figure 5G. Protein–protein interaction network analysis of the top 5 direct binding partners of HNRNPK using STRING database. (**G**) HNRNPK, HNRNPA1 and PTBP1 have binding sites that overlap with TDP-43 binding sites on human *Malat1*. Top: eCLIP read density tracks along human *Malat1*. Boxes underneath each track highlight significant peaks. Black arrow heads indicate peaks that overlap with the human SINE-aligned region. Bottom: Quantification of the percentage of TDP-43 eCLIP peaks that overlap with HNRNPK, HNRNPA and PTBP1’s significant eCLIP peaks.

To determine whether RNAs that carry copies of the SINEB1 of *Malat1* have a tendency for nuclear localization, we interrogated a dataset of RNAs with measured nuclear and cytoplasmic copy numbers in both mouse liver and pancreatic beta cells (36). RNAs with at least one copy of the SINEB1, regardless of whether in the sense or antisense orientation, have a significantly higher ratios of nuclear to cytoplasmic copy numbers than RNAs lacking SINEB1 (Figure 5A). These data further support the cytoplasmic mis-localization phenotype of *Malat1* when the SINEB1 is deleted. Since HNRNPK has previously been shown to drive nuclear retention of RNAs (52), we performed HNRNPK RNA immunoprecipitation (RIP) and observed that HNRNPK binding to SINEB1-deleted *Malat1* significantly decreased (Figure 5B). This may represent the mechanism underlying *Malat1*’s mis-localization to the cytoplasm.

Since the SINEB1 is critical for *Malat1* function and cell survival, we further investigated whether the SINEB1 of *Malat1* is conserved at both DNA and RNA levels. At the DNA level (i.e. genomic), the SINEB1 is only found in mouse but not other mammals including rat and human (Supplementary Figure S6A), whereas the CER region is conserved in all investigated mammals (Supplementary Figure S6B). Although the DNA region of the SINEB1 does not align well in other species’ genomes, the hairpin loop or RBP’s binding site formed or promoted by the SINEB1 may still be conserved through convergent evolution. In fact, regions that are immediately adjacent to the SINEB1 are conserved in many other species but not mice (Supplementary Figure S6A), and may be required for the formation of an essential hairpin loop or RBP binding sites. We speculate that the SINEB1 insertion may have occurred in mice during evolution to possibly recover or gain this essential loop or binding site. To estimate the region of the potential RBP’s binding site formed by the mouse SINEB1 in human Malat1 RNA sequence, we compared *Malat1* cDNA sequences between mouse, human and two other rodents with available *Malat1* cDNA sequences and identified a region that aligned to the SINEB1 region (Supplementary Figure S6C).

Taking advantage of the ENCODE eCLIP-seq database for RBPs in human cell lines, we identified RBPs that bind to the SINE-aligned region in human *Malat1*. Figure 5C shows the top 10 RBPs with the highest number of significant eCLIP peaks that overlap with the SINE-aligned region. In this region KHDRBS1 has the greatest number of significant peaks (Figure 5C). Among these 10 RBPs, three have been experimentally determined to bind directly to HNRNPK, including KHDRBS1, TRA2A and TAF15 (Figure 5D). Of these three RBPs, KHDRBS1 and TRA2A bind predominantly to the 5′ end of human *Malat1*, which encompasses the SINE-aligned region, but not the CER (Figure 5E), suggesting that KHDRBS1 and TRA2A binding to *Malat1* is more likely to be affected by deletion of the SINEB1 rather than the CER region.

**Figure 6.**
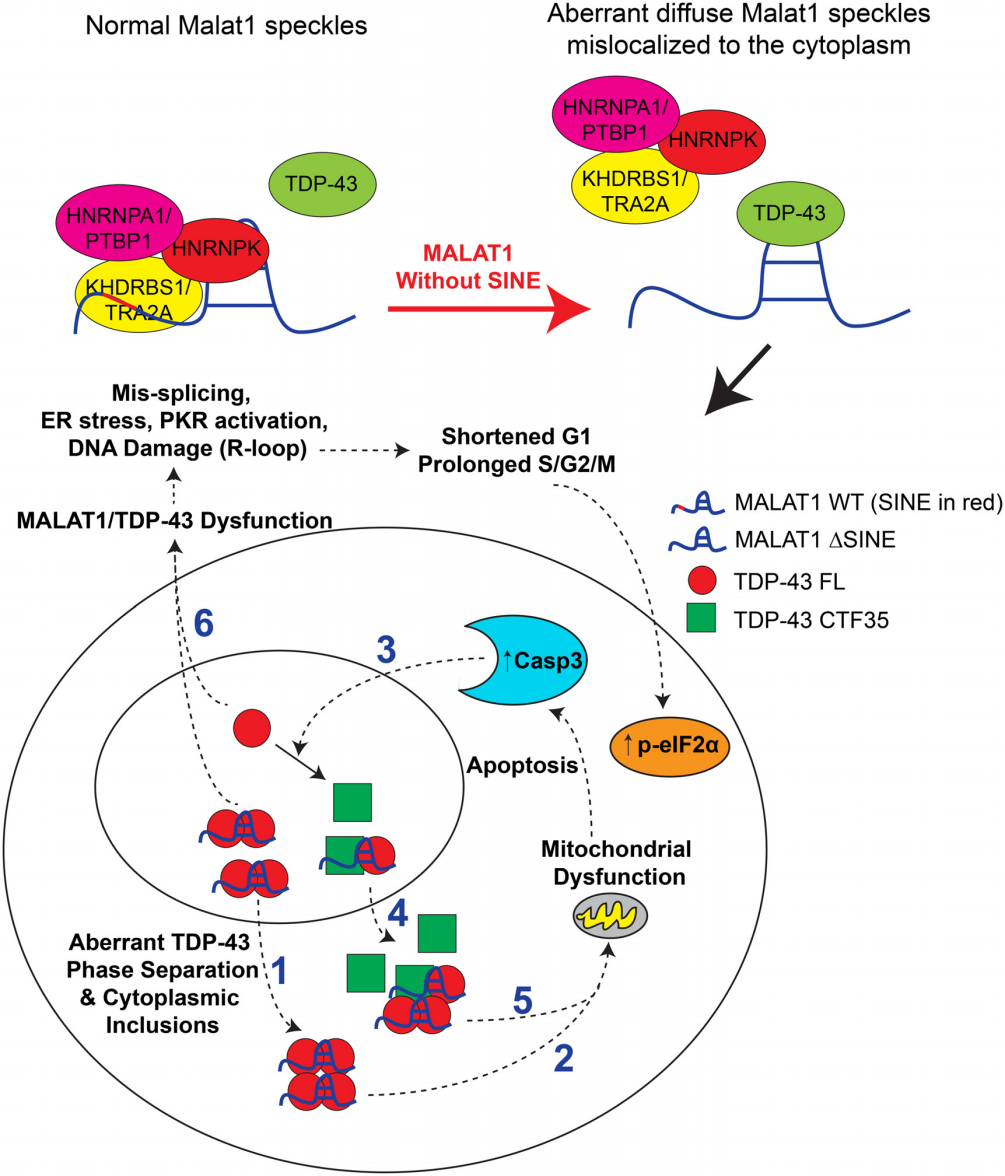
Proposed Model for Malat1 regulation of TDP-43 proteostasis through the SINEB1. In normal cells with the SINE, *Malat1* is bound to HNRNPK, which binds to Malat1 in complexes together with other nuclear-speckle-localized RBPs known to interact with HNRNPK such as HNRNPA1/PTBP1 and KHDRBS1/TRA2A. In the absence of the SINE, *Malat1* loses binding to HNRNPK likely through dissociation of anchor RBPs such as KHDRBS1/TRA2A, which bind predominantly to the SINE region. Note that HNRNPK has been shown to bind RNA indirectly through forming a super complex with KHDRBS1 (i.e. SAM68) (58). HNRNPK dissociation from *Malat1* generates more binding sites for TDP-43 in the absence of the SINE. Moreover, HNRNPK dissociation causes *Malat1* to mislocalize to the cytoplasm, hijacking TDP-43 to the cytoplasm (1), as implicated by the diffuse *Malat1* speckle phenotype with cytoplasmic localization. Both the increase in binding of *Malat1* to TDP-43 and the cytoplasmic mislocalization of TDP-43 have been observed in neurons with cytotoxic TDP-43 inclusions in ALS patients (39, 57). Cytoplasmic TDP-43 inclusions, known to shut down mitochondrial RNAs’ translation and subsequent mitochondrial fragmentation (42), trigger apoptosis and activate caspase-3 (2). Activated caspase-3 cleaves TDP-43 FL into its truncated form TDP-43 CTF35 (3), that predominantly localizes to the cytoplasm (4) and forms more cytotoxic TDP-43 inclusions (5). TDP-43 truncation causes a reduction in nuclear TDP-43 FL that is critical for (6) many RNA metabolism processes as well as DNA repair (54), which disrupts the cell cycle, induce ER stress, PKR activation and subsequent eIF2α phosphorylation.

In addition to KHDRBS1 and TRA2A, HNRNPK is known to interact directly with a number of RBPs, including HNRNPA1 and PTBP1 (Figure 5F), which also bind to *Malat1* (Figure 5G). This suggests that decreased HNRNPK binding to *Malat1* ΔSINE may cause decreased binding of HNRNPA1 and PTBP1 to *Malat1* as well. Furthermore, HNRNPK, HNRNPA1 and PTBP1 all have significant eCLIP peaks that overlap with TDP-43 eCLIP peaks (Figure 5G), suggesting that losing binding of *Malat1* to any of these RBPs will expose more available binding sites for TDP-43, thus explaining the increased binding of *Malat1* ΔSINE to TDP-43. It is worth noting HNRNPK does not have any eCLIP peaks that overlap the SINE-aligned region, suggesting that HNRNPK does not bind directly to *Malat1* at the SINEB1 but likely through KHDRBS1 or TRA2A (Figure 5G). Collectively, these results suggest that HNRNPK likely binds to *Malat1* as a protein complex, in which KHDRBS1 and TRA2A bind directly to the SINEB1, and losing *Malat1* binding to these two RBPs due to the SINEB1 deletion causes decreased HNRNPK binding and potentially its interacting RBPs such as HNRNPA1 and PTBP1 (Figure 6). This decreased binding between *Malat1* ΔSINE and other RBPs allows increased TDP-43 binding to *Malat1* ΔSINE for subsequent mis-localization and aggregation.

## DISCUSSION

In this study, we demonstrated the importance of a 148-bp SINEB1 element in *Malat1*, a cancer-associated lncRNA, in cell cycle regulation, cell survival, genomic stability, response to stress and TDP-43 proteostasis. Removal of the SINE caused formation of diffuse *Malat1* speckles and increased cytoplasmic localization of *Malat1*. In contrast, deletion of another fragment of *Malat1*, the CER, that is approximately 11-fold larger than the SINE had no effect on *Malat1* speckle size and even conferred a protective effect against apoptosis and DNA damage. These phenotypic changes observed for *Malat1* ΔSINE were quite unexpected since the SINE is not involved in formation of intramolecular RNA structure. In contrast, the much larger CER is comprised of many *Malat1* regions that form RNA duplexes (Figure 2B). Examples of roles of lncRNA TEs in mRNA decay, translation and chromatin remodeling have been reported (2–5). Nevertheless, this study identified a novel lncRNA TE that is essential for TDP-43 proteostasis.

The *Malat1* ΔSINE causes multiple detrimental effects on cellular physiology including stress responses such as UPR and PKR activation and particularly apoptosis induced by TDP-43 loss of proteostasis. The change in *Malat1* speckle composition and localization likely precedes all observed phenotypes. Since *Malat1* is highly abundant, changes in *Malat1* localization due to the loss of the SINE can alter localization of many *Malat1*-binding proteins. In fact, a recent study has demonstrated the importance of *Malat1* in regulating the localization and organization of proteins and RNAs in nuclear speckles (30). Therefore, it is not surprising that *Malat1* cytoplasmic translocation was correlated with the cytoplasmic export of TDP-43, a protein that directly binds to *Malat1*. Reduced levels of biologically active TDP-43 in the nucleus due to cytoplasmic export, aggregation, cleavage by caspase-3 and increased binding of *Malat1* ΔSINE to TDP-43 subsequently reprogrammed TDP-43 binding to its RNA targets in the nucleus including repetitive element transcripts and those involved in regulation of mitosis and nuclear-cytoplasmic transport. Consistently, TDP-43 loss of function has been shown to alter splicing of genes involved in mitotic cellular pathways (41), which subsequently can change cell cycle progression and result in abnormal mitosis. TDP-43 depletion can also activate PKR and increase dsRNA levels (53), consistent with the PKR induction phenotype in *Malat1* ΔSINE cells, implicating a role for *Malat1* in innate immunity possibly mediated through TDP-43. In terms of genome integrity, both TDP-43 and *Malat1* have been linked to genome maintenance, in which either TDP-43 or *Malat1* depletion promotes DNA damage (54,55). TDP-43 interacts with key DNA-damage response marker proteins such as γ-H2AX and localizes to DNA-damage regions to mediate DNA repair such as prevention of R-loop formation (46,54), whereas Malat1 has recently been shown to directly bind PARP1 to promote DNA repair (56). Therefore, the increase in DNA damage in *Malat1* ΔSINE cells is likely caused by both TDP-43 dependent (i.e. *Malat1* loss of function) and TDP-43 independent (i.e. TDP-43 nuclear depletion caused by *Malat1*-ΔSINE-mediated cytoplasmic export of TDP-43) manners.

In the cytoplasm, TDP-43 ‘hijacked’ by cytoplasmic *Malat1* also can induce mitochondrial dysfunction and fragmentation by blocking mitochondrial RNA translation (42). This is consistent with condensed mitochondrial phenotype observed in ΔSINE cells (Figure 2G). Subsequently, mitochondrial fragmentation can trigger cytochrome c release and increase cleaved-Caspase 3 levels, resulting in the cleavage of TDP-43 FL into CTF35 that forms aggregates, further amplifying cytotoxicity. In fact, the cytoplasmic mislocalization of TDP-43 is frequently observed in neurons with cytotoxic TDP-43 inclusions in amyotrophic lateral sclerosis (ALS) patients (57).

The diffuse speckles observed for *Malat1* ΔSINE can be explained by decreasing binding of *Malat1* to HNRNPK and potentially its interacting RBPs such as KHDRBS1 and HNRNPA1 (Figure 6). HNRNPK has been shown to bind RNA indirectly through forming a super complex with KHDRBS1 (58). Thus, the significance of the SINEB1 is not only to promote RNA nuclear retention through facilitating RNA binding to HNRNPK, but also to maintain *Malat1* speckle composition and *Malat1*-RBP stoichiometry. The latter is essential in preventing formation of TDP-43 inclusions. *Neat1*, similar to *Malat1*, has been shown to form paraspeckles that co-localize with a fraction of TDP-43 FL and CTF35 aggregate-like speckles, and their interactions have been associated with early phases of ALS pathology (59). Furthermore, the loss in *Malat1*-RBP stoichiometry and reduction of HNRNPK binding to *Malat1* ΔSINE may promote CDK2-mediated phosphorylation of HNRNPK, which is required for TDP-43 accumulation in stress granules (60), and if not resolved can progress into large insoluble aggregates. WT Malat1 together with its associated RBPs may potentially shield HNRNPK’s phosphorylation site. Collectively, formation of cytotoxic TDP-43 aggregates may be due to a combination of TDP-43′s cytoplasmic mislocalization and increased HNRNPK phosphorylation potentially mediated by its reduced binding to *Malat1* and *Malat1*’s associated RBPs.

Although the SINEB1 is not conserved in sequence across species, it may be a part of a conserved hairpin loop or RBP’s binding sites that are essential for scaffolding RBPs. Collectively, this study demonstrates that a small deletion of a TE in a lncRNA can alter proteostasis of its interacting proteins and compromises function, thus emphasizing the importance of TEs that are embedded in lncRNAs in the regulation of RNA–protein stoichiometry and homeostasis.

Both *Malat1* and TDP-43 have been implicated in the pathology of many different cancers, particularly breast cancer. *Malat1* is frequently mutated in breast cancer and has important roles in breast cancer metastasis (61,62). Although TDP-43 is well studied in neurodegenerative diseases, it also has been demonstrated to promote aggressive triple-negative breast cancer, regulate mammary gland development as well as cancer-associated microRNAs (63–65). Furthermore, the aggregate-prone 35kD isoform of TDP-43 has been shown to drive apoptosis in breast cancer (66). Therefore, these findings on the link between *Malat1* and TDP-43 function and toxicity in mammary epithelial cells have potential therapeutic implications in breast cancer. Therapeutics targeting *Malat1* may have anti-cancer properties through induction of TDP-43 toxicity and an innate immune response induced by increased dsRNA levels. Likewise, mutations in *Malat1*, similar to the SINE deletion, may promote breast cancer resistance by activating the unfolded protein response adaptation pathway (67). Since this study did not investigate the role of the SINEB1 of *Malat1* in TPD-43 toxicity in neuronal cell lines, the relevance to neurodegeneration will need to be investigated further. However, this study suggests a potential involvement of mutations in lncRNAs in driving TDP-43 pathology, which may have important clinical implications in neurodegenerative diseases as well as cancer.

In conclusion, this study elucidated the importance of the SINEB1 element in *Malat1*, a lncRNA implicated in many diseases from cancer to neurodegeneration. These unexpected findings raise many questions about how changes in the SINE or TE in lncRNAs in general may impact their function, cellular physiology and evolution. The CRISPR strategy in this study provides a framework for studying TEs embedded in a lncRNA without altering the physiological stoichiometry of lncRNA expression (i.e. overexpression of artificial constructs), the majority of which exhibit low abundance.

## DATA AVAILABILITY

The raw eCLIP-seq data have been deposited to the NCBI Gene Expression Omnibus (GEO; https://www.ncbi.nlm.nih.gov/geo/) under accession number GSE141303.

## Supplementary Material

gkz1176_Supplemental_Files
