# Supplementary material for: The SINEB1 element in the long non-coding RNA Malat1 is necessary for TDP-43 proteostasis
Source: Nucleic Acids Res. 2019 Dec 21;48(5):2621–42. doi: 10.1093/nar/gkz1176 (PMC7049706; doi:10.1093/nar/gkz1176)
Supplement: gkz1176_Supplemental_Files [file gkz1176_supplemental_files.zip › Revised Supplementary Information.docx]

**Supplementary Information for**

“**The SINEB1 Element in the Long Non-coding RNA *Malat1* is Necessary for TDP-43 Proteostasis,**” by Tuan M. Nguyen, Elena B. Kabotyanski, Lucas C. Reineke, Jiaofang Shao, Feng Xiong, Joo-Hyung Lee, Julien Dubrulle, Hannah Johnson, Fabio Stossi, Phoebe S. Tsoi, Kyoung-Jae Choi, Alexander G. Ellis, Na Zhao, Jin Cao, Oluwatoyosi Adewunmi, Josephine C. Ferreon, Allan Chris M. Ferreon, Joel R. Neilson, Michael A. Mancini, Xi Chen, Jongchan Kim, Li Ma, Wenbo Li, and Jeffrey M. Rosen**”**

**Supplementary Figure Legends**

**Figure S1** Cytoplasmic and diffuse *Malat1* speckles in different ΔSINE clones, generation of *Malat1* ΔCER cells and demonstration of the increased cytoplasmic export in interphase *Malat1* ΔSINE cells with large nuclei using an in-house algorithm for distinguishing interphase and mitotic cells.

**(a)** Representative images of cells with cytoplasmic *Malat1* but not cytoplasmic *Neat1* in different ΔSINE clones. FISH probes were used to detect *Malat1* and *Neat1* localization (scale bar = 10 μm). Shown is a representative of ≈ 141 cells for each group.

**(b)** The CER harbors *Malat1* regions previously implicated in cytoplasmic localization of *Malat1*. Positions of the SINE (red) and the CER (blue) relative to *Malat1* fragments previously studied in Miyagawa *et al.* (2012). Genomic coordinates of fragments C, E, H, I, J, K and M in the study are shown. *Malat1* localization when the fragments were overexpressed in cells are indicated as C (cytoplasmic), N (nuclear), N+C (both nuclear and cytoplasmic) and S (in nuclear speckles).

**(c)** Generation and validation of ΔCER cells. Upper: Schema of CRISPR strategy for CER deletion. Middle: Gel electrophoresis image screening for a clone with complete CER deletion. Genomic DNA isolated from the clone was amplified with PCR using primers (red arrows) flanking the deleted region. The upper band is approximately 1700-bp larger than the lower band, which corresponds with the length of the CER. Lower: Alignment of Sanger sequencing reads confirming complete CER deletion relative to the reference WT *Malat1* sequence. Red brackets denote exact location of the CER.

**(d)** Custom-made MATLAB script effectively distinguishes interphase from mitotic cells based on DAPI signal intensity and texture. Left: Representative grayscale DAPI images processed by the algorithm to identify mitotic (red circle) and interphase (green circle) cells. Right: FISH images of *Malat1* and *Neat1* corresponding with the grayscale images on the left (scale bar = 10 μm).

**(e)** Interphase cells distinguished by the custom-made MATLAB script have significantly larger nuclei than mitotic cells across cell types. Boxplot comparing nuclear area between interphase and mitotic cells identified by the algorithm. ****P* < 0.001.

**(f)** Interphase ΔSINE cells still have significantly more cytoplasmic *Malat1* than interphase WT cells even after removing ΔSINE cells with nuclear size twice the average nuclear size of mitotic WT cells, demonstrating that interphase ΔSINE cells with large nuclei still have elevated cytoplasmic export of *Malat1* compared with interphas WT cells. Left: Boxplots showing nuclear area of mitotic WT versus interphase WT and ΔSINE cells without any cutoff (top), with mitotic WT’s mean nuclear area as minimal cutoff (middle), and with twice the mitotic WT’s mean nuclear area as minimal cutoff for interphase ΔSINE cells (bottom). Right: Cumulative distribution of the ratio of cytoplasmic over total Malat1 speckle number comparing WT with clones of ΔSINE cells, corresponding with boxplots on the left with and without nuclear size cutoff. WT, ΔSINE52, ΔSINE14 and ΔSINE50 are shown in blue, purple, red, green, respectively. The cytoplasmic *Malat1* ratio was transformed with x^(1/5), where x is the ratio, to approximate normalization for non-normally distributed data. Two different non-parametric tests, Mann-Whitney U test (MW) and Kolmogorov–Smirnov test (KS), were used showing comparable P values.

**(g)** *Malat1* nuclear speckles are more diffuse in all clones of ΔSINE compared with WT and ΔCER cells. FISH images of *Malat1* and *Neat1* across cell groups (scale bar = 5 μm). Shown is a representative of ≈ 200 cells for each group.

**Figure S2** The SINEB1 of *Malat1* is essential for global translation, reduction of PKR activation and proper cell cycle progression.

**(a)** SINE deletion causes a reduction in global translation. Overlay of polysome profiles of WT and ΔSINE cells. Polysome/mono-disome ratios are highlighted in light blue. Shown is a representative of 3 biological replicates.

**(b)** *Malat1* ΔSINE enhances phosphorylation of PKR, a major sensor of dsRNAs. Western blots on lysates from cells in each group. For phosphorylated PKR (p-PKR), cell lysates were immunoprecipitated (IP) with PKR antibodies then immunoblotted (IB) with p-PKR antibodies. Normalized relative changes in band intensity for individual targets were quantified by ImageJ 1.48v and are shown above each blot. Shown is a representative of 3 biological replicates.

**(c)** ΔSINE cells have significantly more dsRNAs than WT and ΔCER cells. Bars comparing dsRNA levels between ΔSINE and ΔCER relative to WT cells. Cells transfected with poly I:C serve as a positive control for dsRNA induction. Cells stained with an antibody that recognizes dsRNAs were analyzed with flow cytometry. ****P* < 0.001; NS, not significant (*P* > 0.05); error bars indicate standard deviation; *n* = 3 biological replicates.

**(d)** *Malat1* ΔSINE causes accumulation of cells in S and G2/M phases. Cell cycle profiles generated using propidium iodide (PI). Shown is a representative of 3 biological replicates.

**(e)** Quantification and comparison of the cell cycle distribution between clones of ΔSINE and ΔCER versus WT cells. ***P* < 0.01; NS, not significant (*P* > 0.05); error bars indicate standard deviation; *n* = 3 biological replicates.

**(f)** ΔSINE induces micronuclei formation. Shown are representatives of ≈ 117 ΔSINE cells with micronuclei marked by red arrows (scale bar = 10 μm).

**(g)** Quantification of percentages of cells with micronuclei. ****P* < 0.001; NS, not significant (*P* > 0.05); error bars indicate standard deviation; *n* = 3 biological replicates.

**(h)** ΔSINE cells of *Malat1* have abnormal mitosis with multiple nuclei aggregated. Left: p-H3 immunofluorescence staining of cells grown to log phase. White arrows indicate abnormal mitotic cells (scale bar = 25 μm).

**(i)** Quantification of proportions of p-H3 positive cells with aggregated nuclei. **P* < 0.01; error bars indicate standard deviation; *n* = 3 biological replicates.

**(j)** SINE deletion increases mitotic catastrophe. Bars comparing the proportion of WT and ΔSINE cells with mitotic catastrophe. Cells with mitotic catastrophe were identified with live imaging of cells stably expressing Fucci reporters. *Pearson’s* chi-squared test was used for comparisons. ***P* < 0.01; n ≈ 258 cells for each group.

**Figure S3** *Malat1* ΔSINE promotes aberrant cytoplasmic export of TDP-43 full length and Caspase-3-induced cleavage of TDP-43 into aggregate-prone C-terminal fragments through induction of apoptosis, and TDP-43 depletion causes DNA damage in WT cells.

**(a)** Live images of groups of WT and ΔSINE cells transiently expressing TDP-43-GFP. The white arrow heads mark cells with TDP-43 inclusions. Note the prevalence of cytoplasmic TDP-43 in ΔSINE cells.

**(b)** Levels of the insoluble TDP-43 CTF35 dramatically increase in ΔSINE cells. Western blots on soluble and insoluble fractions extracted with RIPA/urea fractionation. Cells treated with the proteasome inhibitor (MG132) serve as a positive control for induction of TDP-43 aggregates. Shown is a representative of 3 biological replicates.

**(c)** Caspase inhibition reduces levels of TDP-43 CTF35 in ΔSINE cells. Western blots on lysates from ΔSINE cells treated with DMSO or Z-VAD-FMK. Shown is a representative of 3 biological replicates.

**(d)** Levels of TDP-43 CTF35 increase in both nuclear and cytoplasmic fractions of ΔSINE cells, whereas TDP-43 FL translocates more to the cytoplasm. Western blots on nuclear and cytoplasmic fractions of WT, ΔSINE and ΔCER cells. HDAC2 and HSP90 serve as markers for nuclear and cytoplasmic fractions, respectively. Note the decreased nuclear TDP-43 FL but increased cytoplasmic TDP-43 FL, indicative of TDP-43 shuttling to the cytoplasm from the nucleus. Shown is a representative of 3 biological replicates. L, ladder.

**(e)** TDP-43 knockdown in WT cells has minimal effects on apoptosis and eIF2α phosphorylation but increases DNA damage. Western blots on lysates from WT cells stably expressing scrambled or TDP-43 shRNA. Shown is a representative of 3 biological replicates.

**Figure S4** Depletion of PKR and PERK, although have no effect on eIF2α phosphorylation and DNA damage, marginally alleviated apoptosis, and depletion of TDP-43 is not sufficient to rescue ER stress in ΔSINE cells.

**(a)** and (**b)** PKR and PERK shRNAs reduce PKR and PERK expression, respectively, in SINE-deleted cells. Bars comparing relative mRNA levels of PKR in (A) and PERK in (B) between WT and ΔSINE cells stably expressing shRNA against PKR and/or PERK versus ΔSINE cells expressing scrambled shRNA (shScrb) control. ***P* < 0.01; ****P* < 0.001; NS, not significant (*P* > 0.05); error bars indicate standard deviation; *n* = 3 biological replicates.

**(c)** PERK and PKR knockdown have no effect on levels of eIF2levels of kdown have nDNA damage but marginally alleviate apoptosis. Western blots on lysates from ΔSINE cells with PKR and PERK knockdown alone or in combination compared with shScrb ΔSINE and WT cells. Normalized relative changes in band intensity are shown above each blot. Shown is a representative of 3 biological replicates.

**(d)** and (**e)** PERK and PKR knockdown do not reduce GADD34 and CHOP expression in ΔSINE cells, respectively. Relative GADD34 and CHOP mRNA levels were measured by qPCR. ***P* < 0.01; NS, not significant (*P* > 0.05); error bars indicate standard deviation; *n* = 3 biological replicates.

**(f)** TDP-43 shRNA decreases TDP-43 expression in WT and ΔSINE cells. Relative TDP-43 mRNA levels were measured by qPCR. ***P* < 0.01; ****P* < 0.001; error bars indicate standard deviation; *n* = 3 biological replicates.

**(g)** and **(h)** TDP-43 knockdown does not reduce GADD34 and CHOP expression, respectively, in ΔSINE cells. Relative GADD34 and CHOP mRNA levels were measured by qPCR. NS, not significant (*P* > 0.05); error bars indicate standard deviation; *n* = 3 biological replicates.

**(i)** TDP-43 knockdown does not decrease the XBP1s/XBP1u ratio in ΔSINE cells. Representative gel electrophoresis images showing differences in XBP1s/XBP1u ratios between cells with and without TDP-43 knockdown.

**(j)** Quantification and comparison of XBP1s/XBP1u ratios between cells treated with shScrb and shTDP-43. **P* < 0.05; ***P* < 0.01; NS, not significant (*P* > 0.05); error bars indicate standard deviation; *n* = 3 biological replicates.

**(k)** PERK and PKR knockdown do not reduce levels of CTF35 TDP-43. Western blots on lysates from ΔSINE cells with PKR and PERK knockdown alone or in combination compared with the scramble control. Shown is a representative of 3 biological replicates.

**Figure S5** Genes with WT- and ΔCER-specific TDP-43 binding are not involved in regulation of mitosis or membrane trafficking and *Malat1* SINE alters TDP-43 binding to its motifs and repetitive element transcripts.

**(a)** TDP-43 antibody specifically immunoprecipitated TDP-43 for eCLIP-seq. Western blot analysis of TDP-43 immunoprecipitation compared with IgG control without and with UV crosslinking.

**(b)** and **(c)** Regulation of mitosis and membrane trafficking are not cellular functions enriched in genes with TDP-43 binding sites that are specific for WT and ΔCER cells, respectively. Cellular function enrichment analysis using Metascape.

**(d)** and **(e)** SINE deletion promotes binding of TDP-43 to an intron of *Cdkn1a (p21)*, an essential cell cycle regulator, and an exon of *Atp2b1*, a plasma membrane calcium-transporting ATPase, respectively. Shown is *Cdkn1a* and *Atp2b1* read density. Average enriched folds above SMInput are shown adjacent to significant peaks (P < 0.01). Arrow indicates the region with increased TDP-43 binding.

**(f)** Both UG-rich and AG-rich motifs are significantly enriched in TDP-43 binding sites in ΔSINE cells. *E* values were determined for UG-rich (left) and AG-rich (right) motifs using DREME algorithm. Note that AG-rich motif is not enriched in WT and ΔCER cells.

**(g)** The fractions of reads that mapped to repetitive elements (RepBase) and the rest of the genome (Genome) in SMInput control and eCLIP TDP-43 IP samples across the different genotypes. Note that although the fraction of reads mapped to repetitive elements in SMInput ΔSINE is slightly less than that in SMInput WT, the fraction of reads in TDP-43 IP ΔSINE that mapped to repetitive elements is higher than that in TDP-43 IP WT.

**(h)** Box plot comparing TDP-43 eCLIP RPM fold across genotypes for repetitive families with at least one member enriched (RPMfold > 1) in at least one genotype. Shown are repetitive families with their enriched members. RPMfold was quantified by dividing normalized reads in IP over that of the corresponding input.

**Figure S6** DNA and cDNA alignment of *Malat1* SINE and CER across species.

**(a)** The *Malat1* SINE genomic DNA only exists in the mouse family but not rat, human or any other species. Gene tree showing genomic alignment of the *Malat1* SINE across 56 eutharian mammals using Ensembl 92 database. Black arrow and dashed lines indicate a DNA region that is not conserved in mice but other species.

**(b)** The *Malat1* CER genomic DNA aligns relatively well across mammals. Gene tree showing genomic alignment of the *Malat1* CER across 56 eutharian mammals using Ensembl 92 database.

**(c)** Alignment of a region of Malat1 cDNA that contains the SINEB1 between mouse, human and two other rodents with available *Malat1* cDNA sequences. The darker the blue color the more conserved the nucleotide. Red dashed lines and arrows mark the exact location of the SINEB1.

**Supplementary Video Legends**

**Video S1 Normal cell division in WT cells.**

WT cells stably expressing Fucci reporters were imaged every 30 minutes (scale bar = 40 μm). Black arrow marks the cell of interest.

**Video S2 Abnormal incomplete mitosis with multiple micronuclei in ΔSINE cells.**

ΔSINE cells stably expressing Fucci reporters were imaged every 30 minutes (scale bar = 40 μm). Black arrow marks the cell of interest.

**Video S3 Mitotic catastrophe in ΔSINE cells.**

ΔSINE cells stably expressing Fucci reporters were imaged every 30 minutes (scale bar = 40 μm). Black arrow marks the cell of interest. Note that daughter cells were unable to re-enter cell cycle.

**Supplementary Tables**

**Table S1** Primers.

| **Experiment** | **Species** | **Name** |  | **Sequence (5’ to 3’)** |
| --- | --- | --- | --- | --- |
| PCR screen for SINE and CER deletion | Mouse | SINE | Forward | CGGCCGTTATAAAAATCCTTC |
|  |  |  | Reverse | CAACCTTTTGGCCTCAATCT |
|  |  | CER | Forward | GACTGGAGCTGCCTTTTGTC |
|  |  |  | Reverse | CTGCAGAGAGCCATTTCCTC |
| qPCR | Mouse | *Malat1* 5’ | Forward | GCATTCAGGCAGCGAGAG |
|  |  |  | Reverse | ATTTCCTCGGGCTGAGTCT |
|  |  | *Malat1*  Middle | Forward | TCTTCTATTCTTCGGCTTCCTACT |
|  |  |  | Reverse | AAGCATCTTTAGAAGACAGAAAAGGT |
|  |  | *Malat1* 3’ | Forward | GTGGAAGCAGATTCGTCAGTAG |
|  |  |  | Reverse | AAAGCTAGGGAAAGGCCAAA |
|  |  | *Malat1*  tRNA | Forward | TGCGGTGTCTTTGCTTGA |
|  |  |  | Reverse | CCATACCCAGAGCCTTTAGAAC |
|  |  | GAPDH | Forward | CTGGAGAAACCTGCCAAGTAT |
|  |  |  | Reverse | GAGTTGCTGTTGAAGTCGCAG |
|  |  | HPRT | Forward | TGCTGACCTGCTGGATTACAT |
|  |  |  | Reverse | TTTATGTCCCCCGTTGACTGA |
|  |  | GADD34 | Forward | AGGACCCCGAGATTCCTCTA |
|  |  |  | Reverse | CTTCGATCTCGTGCAAACTG |
|  |  | CHOP | Forward | GTCCCTAGCTTGGCTGACAGA |
|  |  |  | Reverse | TGGAGAGCGAGGGCTTTG |
|  |  | PKR | Forward | TGTCACACGAGTGCATCTGA |
|  |  |  | Reverse | GTCTGCCCAATTTTGCATTT |
|  |  | PERK | Forward | GATGACGACGTGGAACTGC |
|  |  |  | Reverse | TGAAACCAAGGAACCAGACC |
|  |  | TDP-43 | Forward | ATGGAGTCGTATCAACGCTATG |
|  |  |  | Reverse | CATCAATCCCACACAAGGTTTC |
|  |  | pcDNA3.1 SINE overexpression specific | Forward | GGTTAGGCTAGGGATCCGA |
|  |  |  | Reverse | CAGATGGCTGGCAACTAGAA |
|  |  | pcDNA3.1 WT *Malat1* overexpression specific | Forward | GGAAACAGGGAAGATGGAAGT |
|  |  |  | Reverse | TCTTTCTCCAAATACTAGCCTAACC |
| XBP1 splicing assay | Mouse | XBP1 | Forward | ACACGCTTGGGAATGGACAC |
|  |  |  | Reverse | CCATGGGAAGATGTTCTGGG |
| HNRPNPK RIP RT-PCR | Mouse | *Malat1* | Forward | ATGTCTCCATGGGGAATGAG |
|  |  |  | Reverse | TATGCAGCTTTTCATCAGTAGGA |

**Table S2** sgRNAs for CRISPR-mediated deletion of SINE and CER.

| **Target** | **Plasmid Backbone** |  | **Sequence (5’ to 3’)** |
| --- | --- | --- | --- |
| *Malat1* SINE | PX458 | Top | CACCGGATGGAAGTGTTAGGCTAGC |
|  |  | Bottom | AAACGCTAGCCTAACACTTCCATCC |
|  | PX459 | Top | CACCGAAAAAGGTTAGGCTAGTATT |
|  |  | Bottom | AAACAATACTAGCCTAACCTTTTTC |
| *Malat1* CER | PX458 | Top | CACCGGCCGGTCCCTCGAAAGCGTA |
|  |  | Bottom | AAACTACGCTTTCGAGGGACCGGCC |
|  | PX459 | Top | CACCGGCATAGAGGCCGTCTAACTA |
|  |  | Bottom | AAACTAGTTAGACGGCCTCTATGCC |

**Table S3** shRNA targeted sequences.

| **Species** | **Name** | **Targeted Sequence (5’ to 3’)** |
| --- | --- | --- |
| Mouse | PERK | CCATGAGTTCATCTGGAACAA |
|  | PKR | GGAGTAGCCATTACGTATAAA |
|  | Scrb for shPKR and shPERK | CCTAAGGTTAAGTCGCCCTCG |
|  | TDP-43 | CTCTGTACAACGTCCAACA |
|  | Scrb for shTDP-43 | TGGTTTACATGTTGTGTGA |

**Table S4** eCLIP-seq oligos.

| **Name** | **Sequence (5’ to 3’)** |
| --- | --- |
| RiL19 | /5phos/rArGrArUrCrGrGrArArGrArGrCrGrUrCrGrUrG/3SpC3/ |
| rand103Tr3 | /5Phos/NNNNNNNNNNAGATCGGAAGAGCACACGTCTG/3SpC3/ |
| PCR_F_D501 | AATGATACGGCGACCACCGAGATCTACACTATAGCCTACACTCTTTCCCTACACGACGCTCTTCCGATCT |
| PCR_R_D701 | CAAGCAGAAGACGGCATACGAGATXXXXXXXXGTGACTGGAGTTCAGACGTGTGCTCTTCCGATC |
