# Supplementary figures and images for: The SINEB1 element in the long non-coding RNA Malat1 is necessary for TDP-43 proteostasis
Source: Nucleic Acids Res. 2019 Dec 21;48(5):2621–42. doi: 10.1093/nar/gkz1176 (PMC7049706; doi:10.1093/nar/gkz1176)

# Figure S2

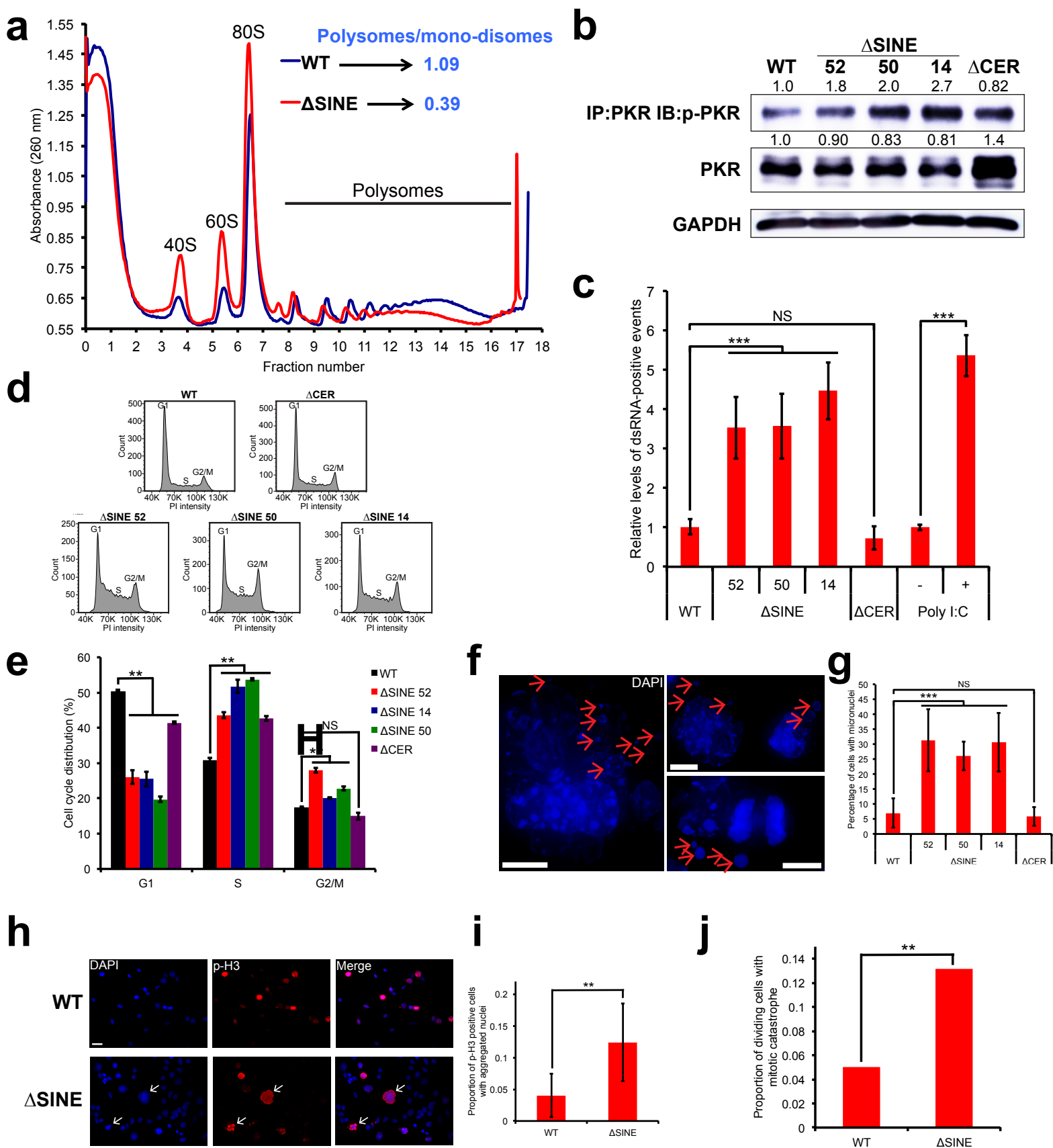

# Figure S3

**a**

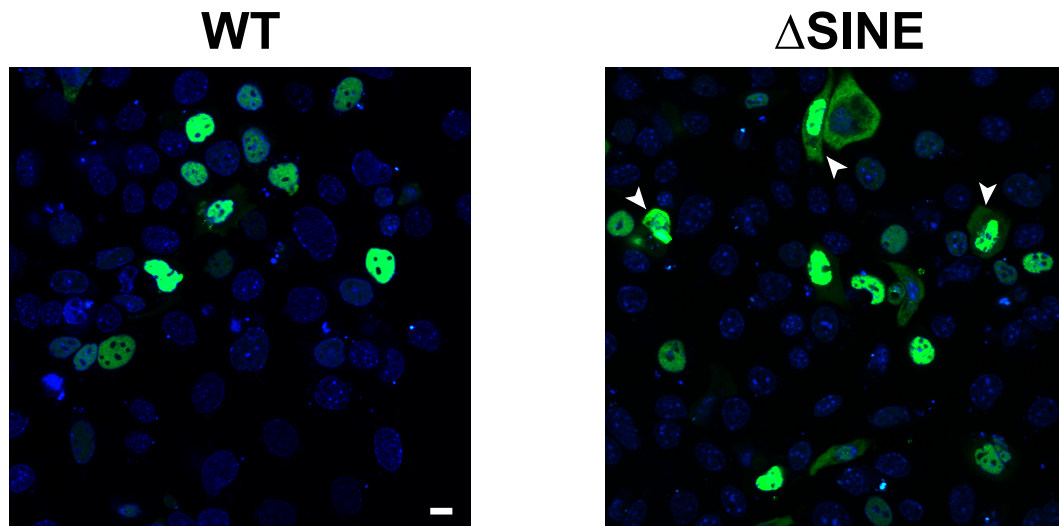

**b**

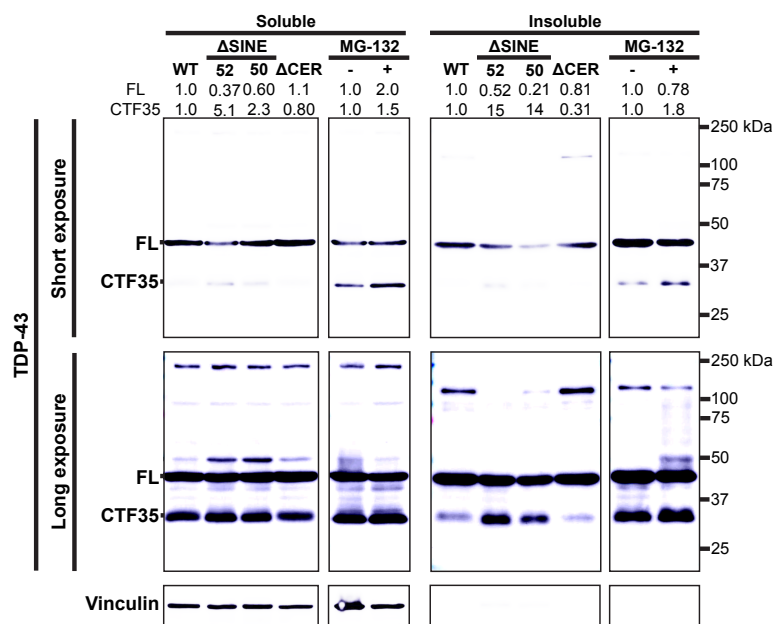

**c**

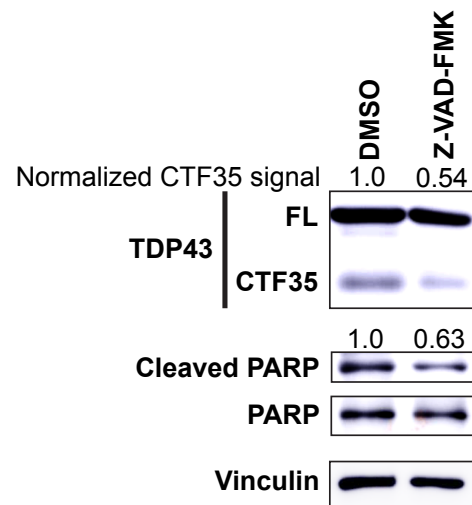

**d**

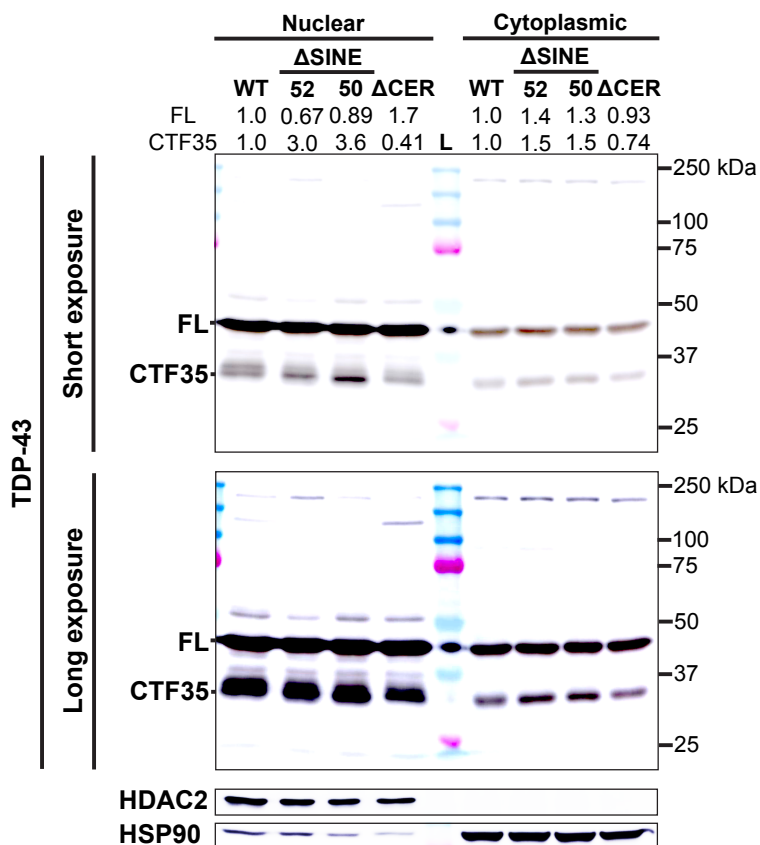

**e**

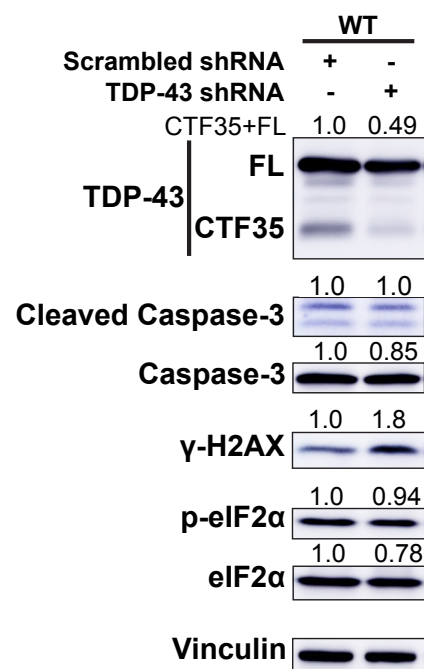

# Figure S4

**a**

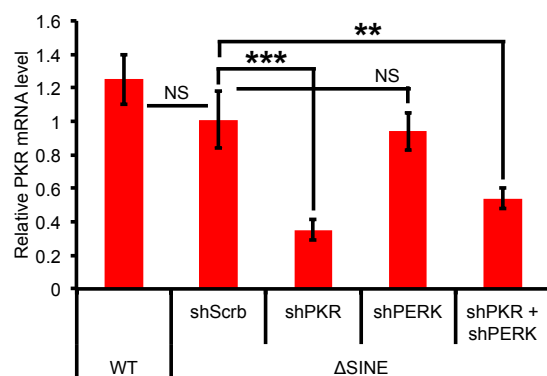

**b**

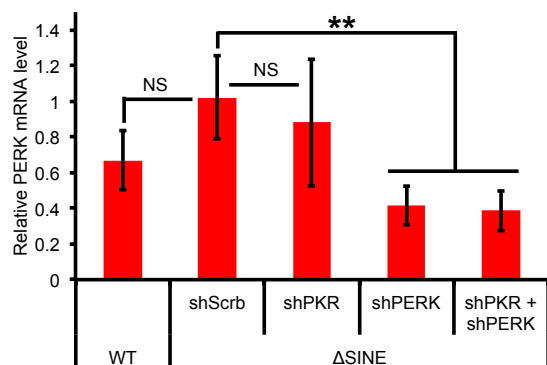

**d**

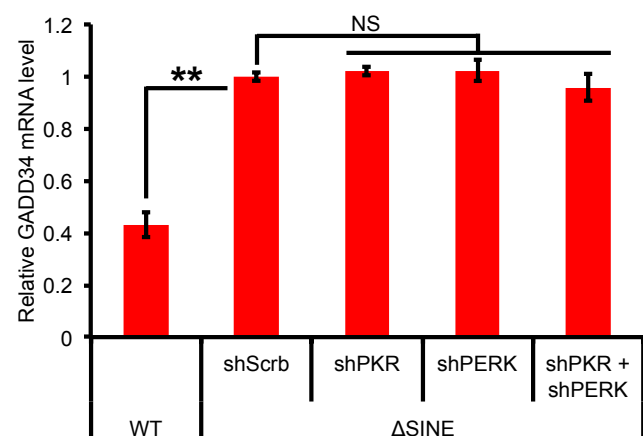

**c**

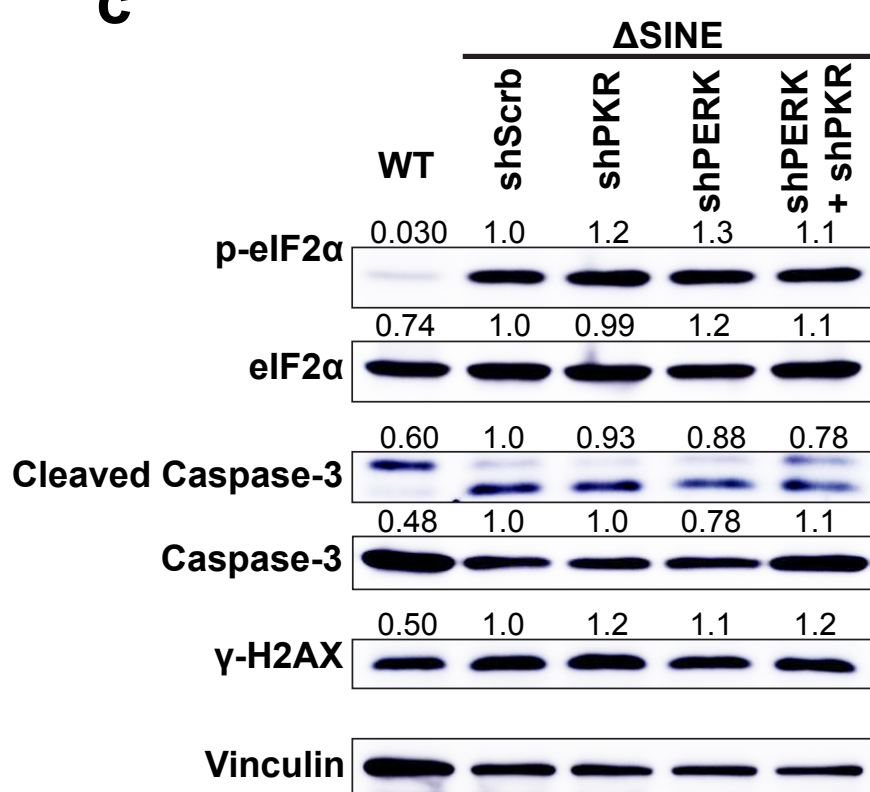

**e**

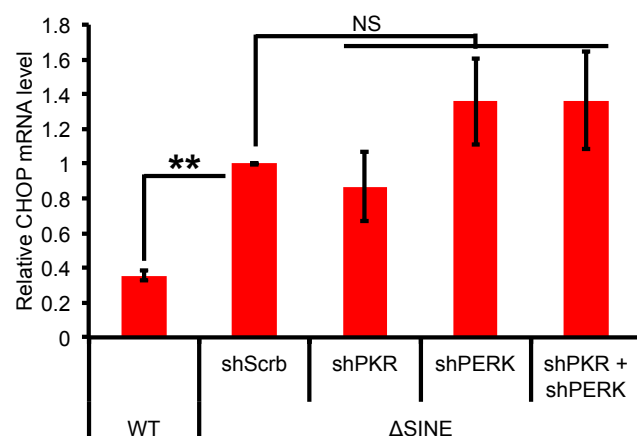

**f**

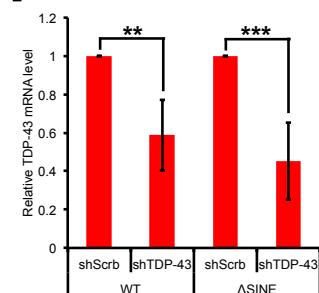

**g**

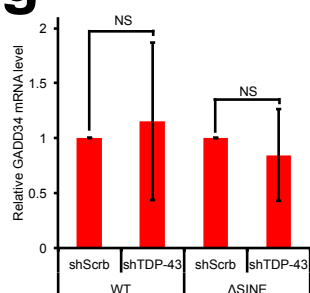

**h**

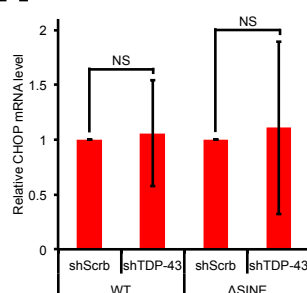

**k**

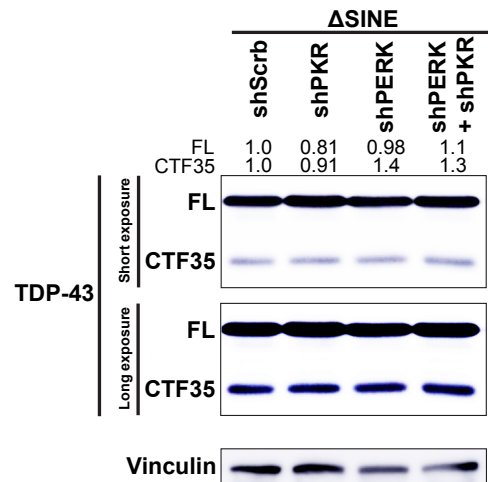

**i**

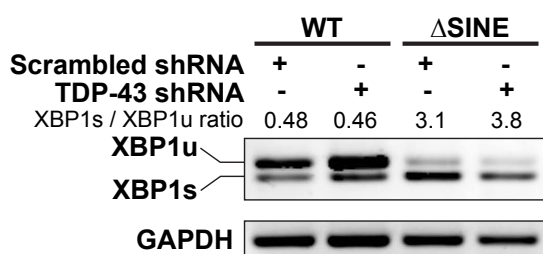

**j**

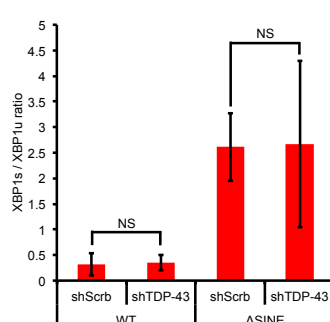

# Figure S5

a

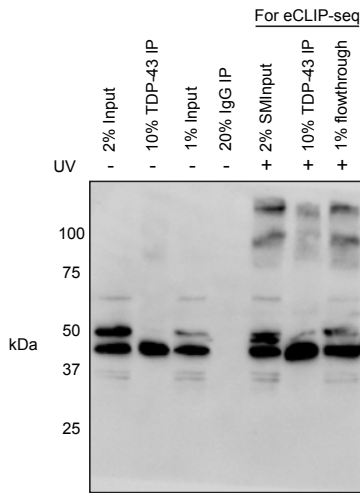

b

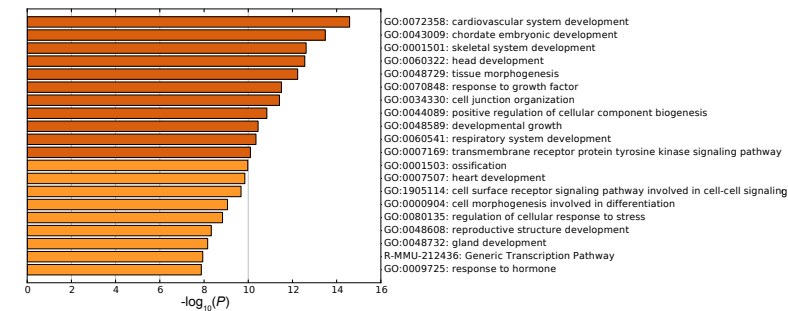

c

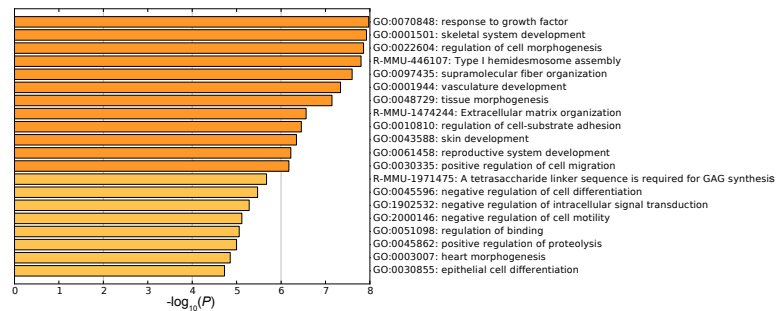

d

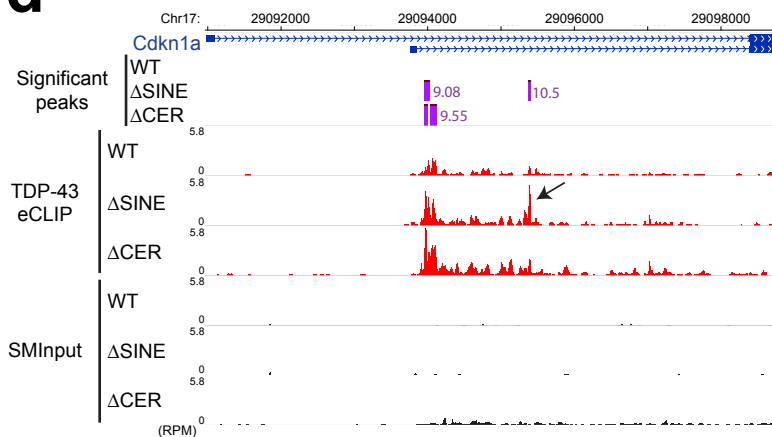

e

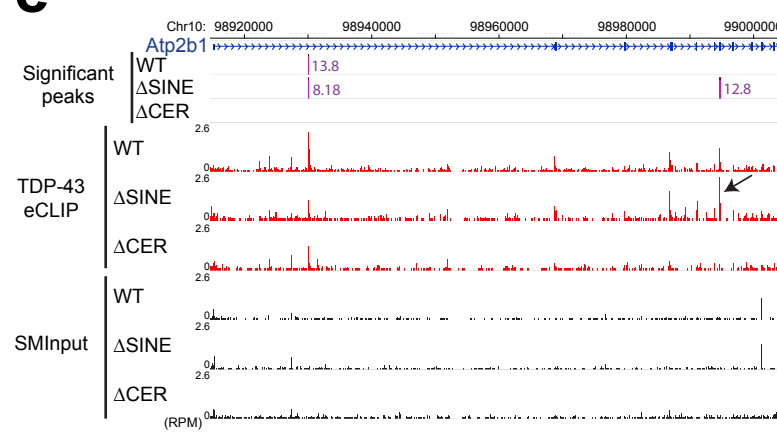

f

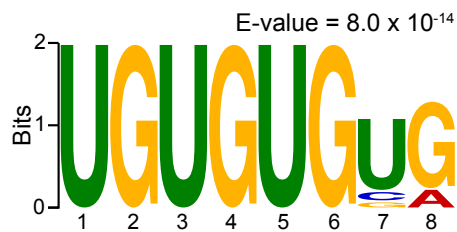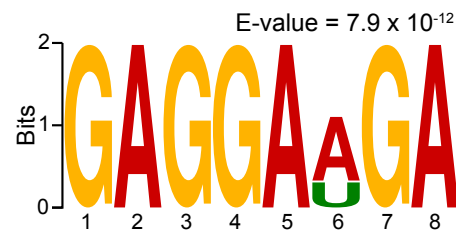

g

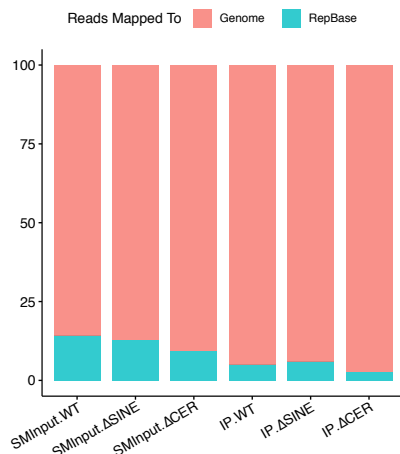

h

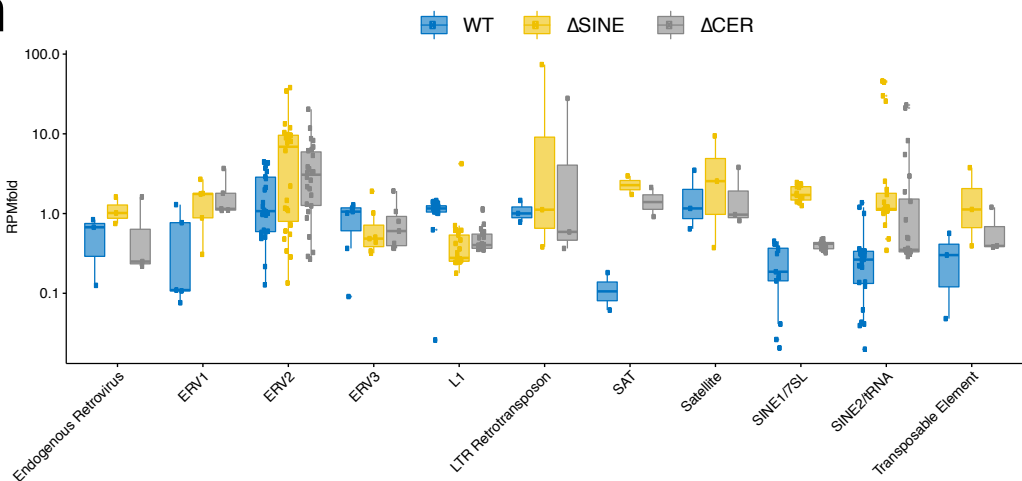

Figure S6

a

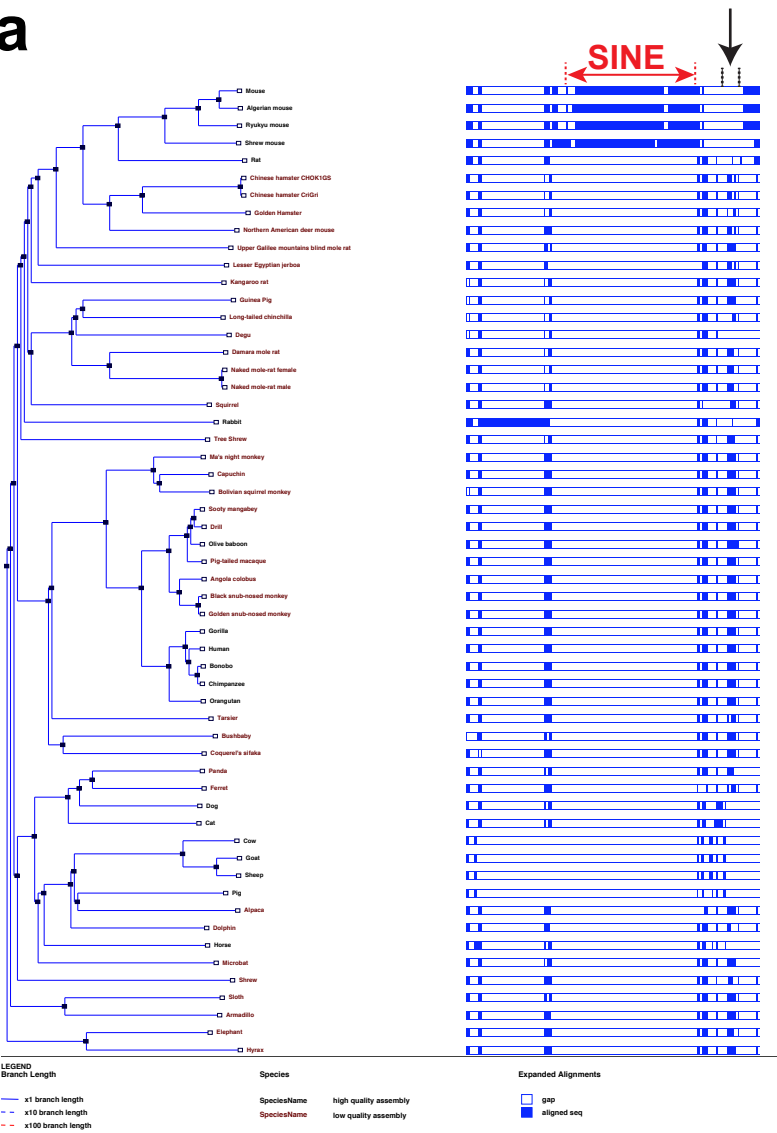

b

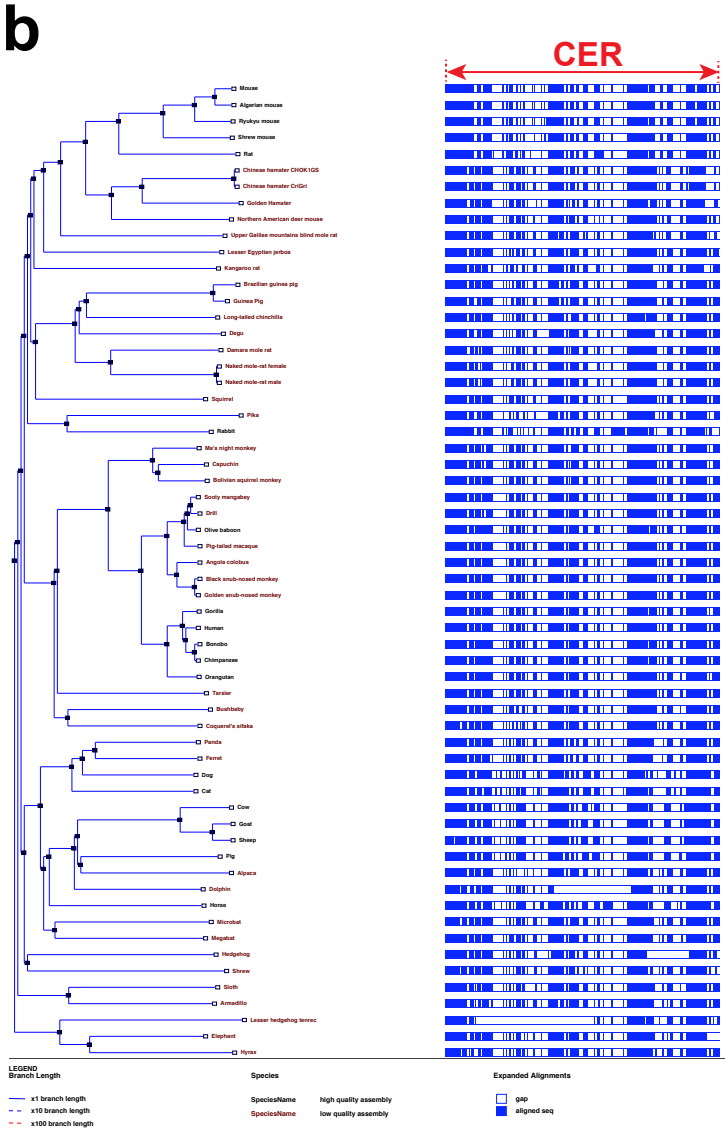

c

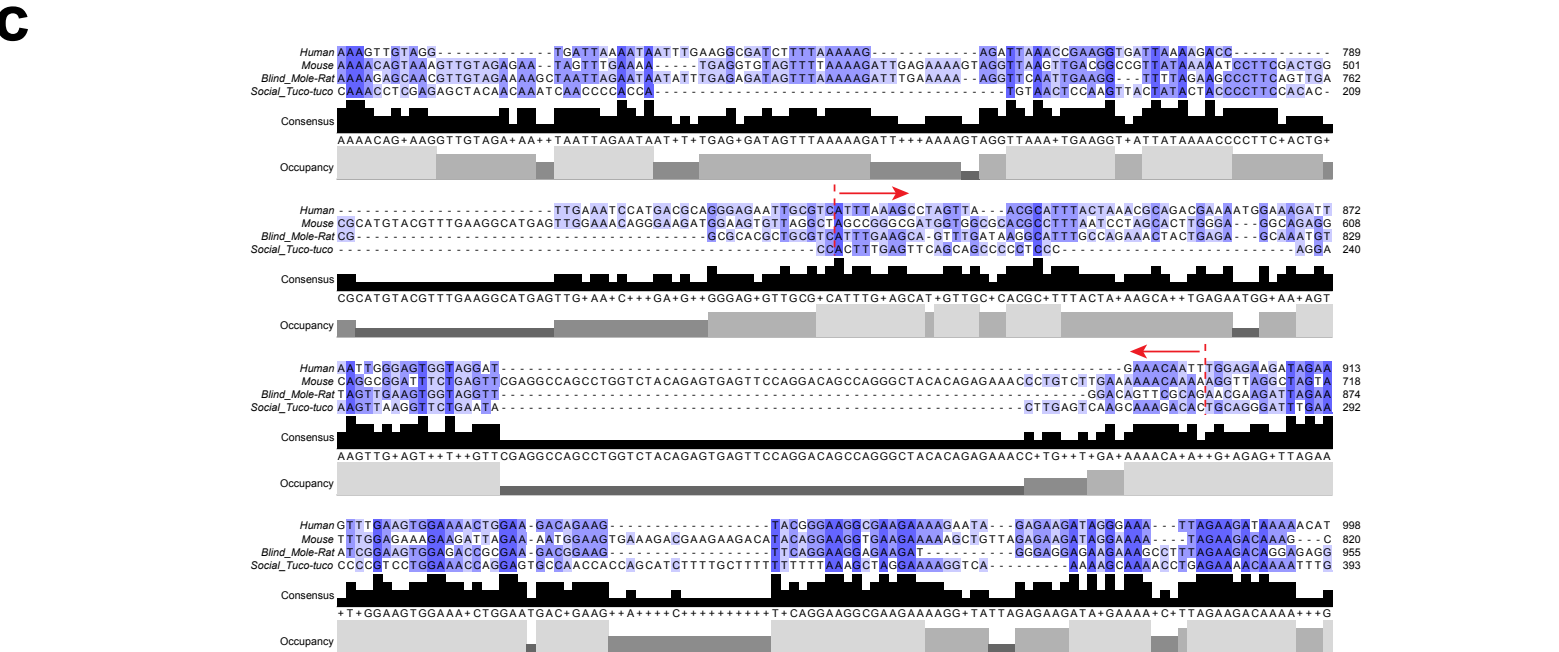

Supplement: gkz1176_Supplemental_Files [file gkz1176_supplemental_files.zip › Supplementary_Figures.pdf]
